# LMO4 promotes OSCC progression by inducing RAB17 degradation and ferroptosis resistance

**DOI:** 10.1038/s41419-025-08171-1

**Published:** 2025-11-10

**Authors:** JiaJia Fan, Hongyan Zhang, Lin Liu, Chunyu Wang, Shiheng Jia, Qian Wang, Zengyan Xu, Fengfei Zhao, Shuzhen Xiang, Wei Ma, Zhuoran Huang, Minda Liu, Yanshu Li, Wei Dai

**Affiliations:** 1 Department of Oral Maxillofacial-Head and Neck Surgery, School of Stomatology, China Medical University, Oral Diseases Laboratory of Liaoning, 117 Nanjing North Road, Heping District, Shenyang, Liaoning 110000 China; 2 Department of Cell Biology, Key Laboratory of Cell Biology, Ministry of Public Health, and Key Laboratory of Medical Cell Biology, Ministry of Education, China Medical University, Shenyang, Liaoning 110122 China; 3 Department of Hematology, The Fourth Affiliated Hospital of China Medical University, Shenyang, 110033 China; 4 Department of Surgical Oncology and General Surgery, The First Hospital of China Medical University, Shenyang, Liaoning 110001, China; Key Laboratory of Molecular Pathology and Epidemiology of Gastric Cancer in the Universities of Liaoning Province, Shenyang, Liaoning 110001 China; 5 School of Stomatology, China Medical University, Liaoning Shenyang, 110122 China; 6 Department of Clinical Medicine, China Medical University, Liaoning Shenyang, China

**Keywords:** Oral cancer, Extracellular signalling molecules, Post-translational modifications, Cancer genomics, Cancer

## Abstract

Oral squamous cell carcinoma (OSCC) is an aggressive cancer with limited improvement in patient outcomes despite advances in surgery, chemotherapy, and radiotherapy. The LIM-only protein LMO4 functions as a transcriptional co-regulator and is known to be increased in several epithelial cancers, but its contribution to OSCC has not been well defined. In this study, we found that LMO4 expression was markedly higher in OSCC tissues and was associated with poorer overall survival. Cellular experiments showed that LMO4 enhanced OSCC cell proliferation, migration, and resistance to ferroptosis by promoting the ubiquitin–proteasome–dependent degradation of the tumor suppressor RAB17. Restoration of RAB17 expression reduced these malignant behaviors. In a nude mouse xenograft model, tumors with high LMO4 grew faster and displayed lower RAB17 protein levels. Taken together, our results indicate that LMO4 contributes to OSCC progression through post-translational regulation of RAB17 and ferroptosis control, suggesting that this pathway could serve as a new therapeutic target.

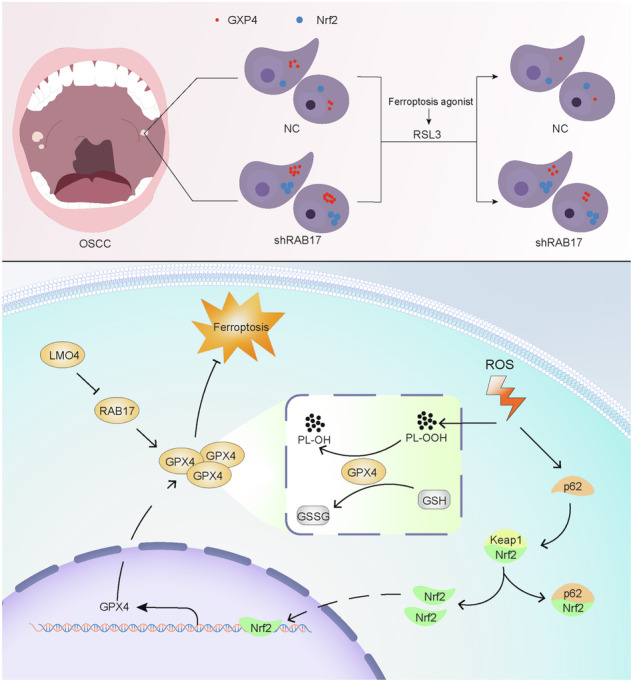

## Abstract (web summary)

RAB17 and LMO4 modulate ferroptosis by regulating GPX4 expression through the Nrf2-Keap1 signaling pathway.

## Introduction

Oral squamous cell carcinoma (OSCC) is the most common type of oropharyngeal cancer [1], characterized by high malignancy and poor prognosis. Although surgical resection combined with chemotherapy and radiotherapy is applied in many cases, the 5-year survival rate of OSCC patients has not exceeded 50% over the past two decades [2]. Metastasis is particularly fatal, often leading to mortality within one year of diagnosis in advanced cases [3]. Therefore, investigating the molecular mechanisms underlying OSCC progression and metastasis is vital for identifying new therapeutic targets.

LMO4 (LIM domain only protein 4) is a small transcriptional regulator composed of two tandem LIM domains that mediate protein–protein interactions [4]. Initially identified as an oncogene in T-cell leukemia and subsequently detected in breast cancer [5], LMO4 modulates tumorigenesis, apoptosis, and hormone signaling [6]. It enhances invasion and migration in gastric cancer and modulates IL-23 signaling in psoriasis [7], and promotes CD8 + T cell stemness to improve immunotherapy efficacy [8]. In head and neck squamous cell carcinoma (HNSCC), LMO4 is overexpressed at tumor invasion fronts and in lymph node metastases [9], where it interacts with LIM domain-binding protein 1 (LDB1) and shows enhanced nuclear localization [10].

RAB17 (Ras-related protein Rab-17), a member of the RAB GTPase family, regulates intracellular trafficking and contributes to tumor development [11]. It was initially discovered in epithelial cells during polarization in mouse kidney tissue [12] and later found in the cytoplasm where it directs transport processes [13]. In various cancers, RAB17 acts context-dependently: it is inhibited by ERK2 (Extracellular Signal-Regulated Kinase 2) to facilitate breast cancer cell migration [14], suppresses hepatocellular carcinoma progression [15], and contributes to paclitaxel resistance in ovarian cancer [16]. In non-small cell lung cancer, its downregulation promotes proliferation, migration, and epithelial-mesenchymal transition (EMT) [17].

Post-translational modifications (PTMs), such as ubiquitination, are crucial for protein homeostasis. The ubiquitin–proteasome system (UPS) degrades over 80% of cellular proteins [18], and in cancer, aberrant UPS activity may destabilize tumor-suppressor complexes [19]. Ubiquitination involves E1, E2, and E3 enzymes that sequentially tag proteins for proteasomal degradation [20]. Ferroptosis, an iron-dependent, non-apoptotic cell death pathway triggered by lipid peroxidation and ROS (Reactive Oxygen Species) accumulation [21], has emerged as a key regulator in cancer biology [22]. It is induced by inhibition of xCT (SLC7A11, a cystine/glutamate antiporter) or glutathione peroxidase 4 (GPX4), leading to glutathione depletion and oxidative damage [23].

In this study, we constructed LMO4-overexpressing and knockdown OSCC cell lines to investigate the role of LMO4 in OSCC progression. Functional assays were used to assess effects on proliferation, migration, and ferroptosis. Proteomic analysis identified RAB17 as a novel downstream effector regulated by LMO4 through ubiquitin-mediated degradation, offering mechanistic insight and potential therapeutic implications for targeting the LMO4-RAB17 axis in OSCC.

## Materials and methods

### Cell culture

The HaCaT cell line (human keratinocyte) was purchased from Beyotime Biotechnology (C6282, Shanghai, China). CAL27 (ATCC® CRL-2095™), and SCC-9 (ATCC® CRL-1629™) cell lines were obtained from the American Type Culture Collection (ATCC, Manassas, VA, USA), while HSC-2 cells were sourced from Pricella Biological Technology (CL-0884, Wuhan, China). HSC-4 cells (CVCL_1289, Osaka, Japan) were also acquired from JCRB. CAL27, HSC-4, SCC-9, and HSC-2 cells were cultured in Dulbecco’s Modified Eagle Medium (DMEM, Gibco, USA) supplemented with 10% fetal bovine serum (FBS, Gibco, USA) and 1% penicillin-streptomycin (KGI, Nanjing, China). All cultures were maintained at 37 °C with 5% CO₂ in a humidified incubator.

### Plasmids and cell line construction

LMO4-overexpressing (LMO4-OE) cell lines were generated by transducing cells with lentiviral plasmids in which the full-length human LMO4 cDNA was inserted into the GV141 backbone (GV141-LMO4) (Fig. S1A). LMO4-knockdown (shLMO4) cell lines were established using GV248-based lentiviral plasmids carrying three independent shRNA sequences targeting (Fig. S1B). For LMO4 knockdown, three shRNA sequences were used: 1# 5’-caCTACATCAATGGCAGTTTA-3’, 2# 5’-gcGCAAGGCAATGTGTATCAT-3’, and 3# 5’-caGAAATGACTACATTAGGTT-3’; the negative control (NC) sequence was 5’-TTCTCCGAACGTGTCACGT-3’ (all from Genechem, Shanghai, China). Lentiviral particles were produced using HEK293T cells (the Cell Bank of the Chinese Academy of Sciences, Shanghai, China), and infection was performed with 8 μg/mL polybrene (GeneChem, Shanghai, China). Puromycin (2 μg/mL, purchased from Selleck Chemicals, Shanghai, China) selection was applied for 7–10 days to establish stable cell lines. Empty vector was used as control (GV141 and GV248). Efficiency was confirmed by qRT-PCR (quantitative real-time polymerase chain reaction) and Western blot.

### Human tissue samples

Fresh-frozen tumor tissues and matched adjacent normal oral mucosa were obtained from patients diagnosed with OSCC who underwent surgical resection at the School and Hospital of Stomatology, China Medical University, between March 2023 and September 2023. The inclusion criteria were: (1) histopathologically confirmed primary OSCC, (2) no prior radiotherapy, chemotherapy, or immunotherapy before surgery, and (3) availability of matched adjacent normal tissue. Patients with recurrent OSCC, other malignancies, or severe systemic diseases were excluded. Adjacent normal tissues were collected at least 2 cm away from the tumor margin and histologically confirmed to be free of tumor cells. All specimens were collected under the approval of the Ethics Committee of the School of Stomatology, China Medical University (approval no. 2023-CMU-027; date: 7 March 2023) and with written informed consent from all participants. Samples were immediately snap-frozen in liquid nitrogen and stored at −80 °C until analysis. A portion of each specimen was fixed in 10% neutral-buffered formalin, embedded in paraffin, and used for immunohistochemistry (IHC) analysis. Clinicopathological information was obtained from medical records.

### Protein extraction and western blot

Proteins were extracted using RIPA buffer (Beyotime, P0013B) supplemented with protease and phosphatase inhibitors, quantified with the BCA protein assay kit (Beyotime, P0010), and separated by SDS-PAGE. For all Western blot experiments, 20 μg of total protein was loaded per lane, and the same amount was used for both experimental and control groups to ensure consistency. After transfer to PVDF membranes (Millipore, USA), membranes were blocked with 5% skimmed milk, incubated with primary antibodies (e.g., anti-LMO4, anti-RAB17, anti-ubiquitin, anti-β-actin, specific product codes and manufacturers provided in Supplementary Table S1), followed by HRP-conjugated secondary antibodies (Cell Signaling Technology, Danvers, MA, USA). Detection was performed using ECL chemiluminescence reagents (Tanon, Shanghai, China), and signals were visualized using the Tanon 4600 imaging system and analyzed with Tanon Gel Imaging Software (Shanghai Tanon Science & Technology Co., Ltd., Shanghai, China). Band intensity was quantified using ImageJ.

### Quantitative real-time PCR (qRT-PCR)

Total RNA was extracted using RNAiso Plus (Takara, Japan, Cat. No. 9108) according to the manufacturer’s protocol. Complementary DNA (cDNA) was synthesized from 1 µg of total RNA using the PrimeScript™ RT Reagent Kit (Takara, Japan, Cat. No. RR037A). Quantitative real-time PCR was conducted using TB Green® Premix Ex Taq™ II (Takara, Japan, Cat. No. RR820A) on an Agilent MX3000P Real-Time PCR System (Agilent Technologies, USA). GAPDH was used as internal control. Relative gene expression was calculated using the 2^–ΔΔCt method. Primer sequences are provided in Supplementary Table S2.

### Mass spectrometry-based proteomic analysis

LMO4-overexpressing CAL27 cells and corresponding control cells were lysed in RIPA buffer (Beyotime, P0013B) supplemented with protease inhibitors (Roche, 04693159001). Protein concentrations were determined with a BCA assay (Thermo Fisher, 23227). For each sample, 100 μg of total protein was used for tryptic digestion with sequencing-grade trypsin (Promega, V5111) following the FASP (Filter-Aided Sample Preparation) protocol. Peptides were desalted on C18 columns (Waters, WAT054955) and subjected to LC-MS/MS (liquid chromatography-tandem mass spectrometry) analysis using a Q Exactive Plus Orbitrap mass spectrometer (Thermo Fisher Scientific) at BPI Biotech (Beijing, China). MS data were processed with MaxQuant (v1.6.17.0). Label-free quantification (LFQ) was performed. Differentially expressed proteins were defined by fold change >1.5 or <0.67 and *p* < 0.05.

### Immunohistochemistry and survival analysis

Tumor tissue sections were deparaffinized, rehydrated, and subjected to antigen retrieval using citrate buffer (pH 6.0) in a pressure cooker for 2 min. Endogenous peroxidase activity was blocked with 3% hydrogen peroxide. Slides were incubated with primary antibodies against LMO4 and RAB17 overnight at 4°C, followed by HRP-labeled secondary antibodies and developed with DAB (ZSGB-BIO, Beijing, China). Hematoxylin was used for counterstaining. Staining intensity and percentage were scored; scores 0-4 was defined as low expression, and 6-12 as high expression. Survival analysis was conducted using the Kaplan-Meier method.

### Confocal microscopy

Cells grown on coverslips were fixed with 95% ethanol, permeabilized with 0.1% Triton X-100, and blocked with 5% goat serum. Primary antibodies were incubated overnight at 4 °C, followed by Alexa Fluor 488 or 594-conjugated secondary antibodies (1:200 dilution, Thermo Fisher). Nuclei were stained with DAPI (4’,6-diamidino-2-phenylindole). Images were acquired using an Opera Phenix high-content live-cell imaging system (PerkinElmer, USA).

### EdU proliferation assay

EdU (5-ethynyl-2’-deoxyuridine) incorporation was assessed using the Cell-Light™ EdU Apollo567 In Vitro Kit (RiboBio, Guangzhou, China). Cells were seeded into 24-well plates at a density of 5 × 10⁴ cells/well, labeled with 50 µM EdU for 2 h, fixed, permeabilized, and stained with Apollo dye and Hoechst 33342. Images were captured using a fluorescent microscope.

### CCK-8 assay

Cell proliferation was measured using the Cell Counting Kit-8 (CCK-8, Dojindo, Japan). Log-phase cells were seeded in 96-well plates at a density of 2 × 10^3^ cells/well, and absorbance at 450 nm was measured after incubation with 10 µL reagent for 1.5 h.

### Colony formation assay

Log-phase cells were seeded into six-well plates (100 cells/well) and cultured for 10–15 days. Colonies were fixed with 95% ethanol for 30 min, stained with 0.1% crystal violet for 2 h, washed with distilled water, and photographed for statistical analysis. In general, the overexpression group was cultured for 10 days, while the knockdown group required 15 days to allow visible colony formation.

### Wound healing assay

Cells were grown to 90% confluency, and wounds were scratched with a pipette tip. After PBS washing, cells were cultured in serum-free medium. Images were captured at 0 and 24-48 h, and wound closure was quantified.

### Transwell migration assay

Migration was assessed using Transwell chambers (8 μm pore, Corning, USA). Cells were seeded in the upper chamber in serum-free DMEM, while the lower chamber contained 20% FBS. For the Con/LMO4-OE and NC/si-RAB17 groups, cells were seeded at a density of 4 × 10⁴ cells/well, whereas for the NC/si-LMO4 and Con/RAB17-OE groups, the seeding density was 1 × 10⁵ cells/well. After incubation, migrated cells were fixed, stained with crystal violet, and counted.

### Bioinformatics analysis

Transcriptomic data related to RAB17 expression in head and neck squamous cell carcinoma (HNSC) were obtained from publicly available databases. Gene expression profiles were analyzed using the GEPIA2 platform (http://gepia2.cancer-pku.cn), accessed in January 2024, and the UALCAN portal (https://ualcan.path.uab.edu/cgi-bin/ualcan-res.pl).

### Transmission electron microscopy (TEM)

Cells were fixed in 2.5% glutaraldehyde at 4 °C overnight and post-fixed in 1% osmium tetroxide for 1 h. After dehydration through a graded ethanol series, the samples were embedded in epoxy resin. Ultrathin sections (~70 nm) were prepared using an ultramicrotome, stained with uranyl acetate and lead citrate, and examined under a Hitachi H-7650 transmission electron microscope (Hitachi High-Technologies, Japan).

### In vivo tumorigenicity assay

All animal procedures were approved by the Animal Ethics Committee of China Medical University and conducted in accordance with institutional guidelines. Mice were randomly assigned to experimental or control groups before treatment. CAL27 cells were allocated into four groups: GV141 (control, 1×10^6^ cells/100 μL), LMO4 overexpression (1×10^6^ cells/100 μL), GV248 (negative control, 2 ×10^6^ cells/100 μL), and LMO4 knockdown (2 ×10^6^ cells/100 μL). Each cell suspension was mixed with Matrigel (Corning; 7:3 ratio) and subcutaneously injected into the right axilla of 5-6-week-old female BALB/c-nude mice (n = 5 per group; Vital River, Beijing, China). All animals meeting the experimental criteria were included in the analysis. Tumor dimensions were measured every three days with digital calipers, and tumor volume was calculated using the formula: Volume = (length × width²) / 2. Mice were euthanized when tumor volume reached 1.0 cm^3^, and tumors were excised for protein extraction and histological analysis. Sample size followed previous OSCC xenograft studies; mice were randomly assigned to groups before treatment.

### Statistical analysis

Statistical analysis was performed using GraphPad Prism (v9.0.0.121, GraphPad Software, San Diego, CA, USA) and ImageJ (v1.x, National Institutes of Health, Bethesda, MD, USA). Results are expressed as mean ± SD. Comparisons between two groups used Student’s t-test or Mann–Whitney U test; comparisons among three or more groups used one-way ANOVA or Kruskal–Wallis test. Kaplan–Meier curves were analyzed using the log-rank test. *p* < 0.05 was considered statistically significant (*p < 0.05, **p < 0.01, *p < 0.001, ns: not significant).

## Results

### Upregulation of LMO4 in OSCC tissues and its correlation with poor prognosis

**Fig. 1:**
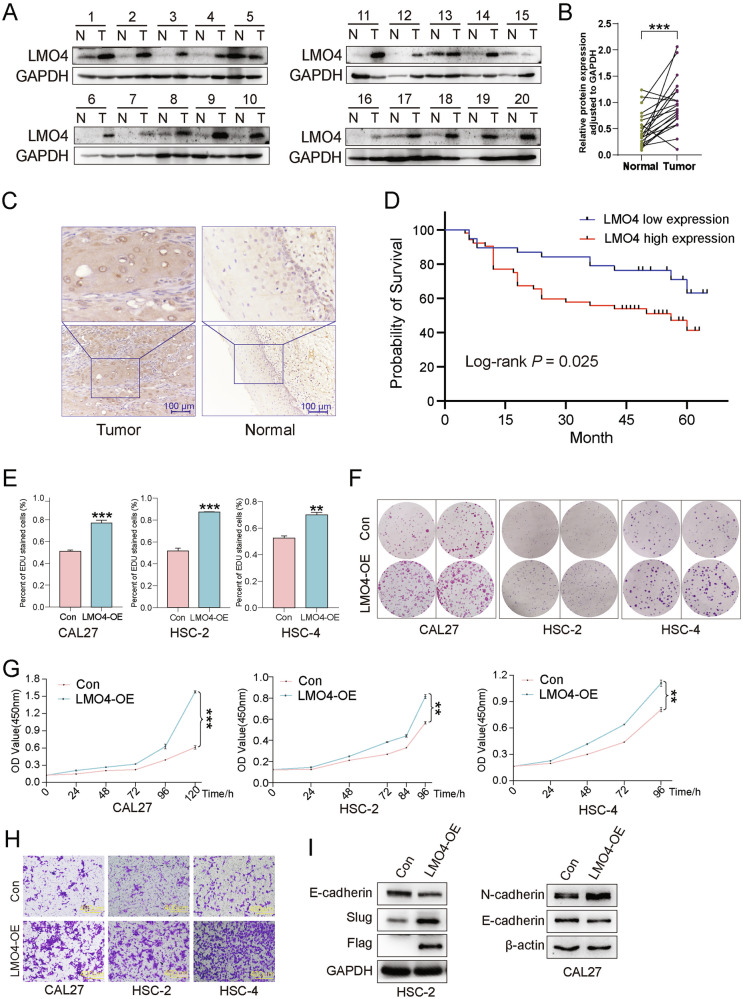
LMO4 is upregulated in OSCC tissues and correlates with poor prognosis. **A** Western blot analysis showing the expression of LMO4 in paired normal (N) and tumor (T) tissues from 20 OSCC patients. GAPDH was used as a loading control. **B** Quantification of LMO4 protein expression in normal and tumor tissues. Data are normalized to GAPDH and presented as relative protein expression levels. **C** Representative immunohistochemical (IHC) staining of LMO4 in OSCC tumor tissues and adjacent normal tissues. Magnified views of the boxed areas are shown below. Scale bars = 100 μm (top), 200 μm (bottom). **D** Kaplan-Meier survival curves comparing the overall survival of OSCC patients with high and low LMO4 expressions. Log-rank test, *p* = *0.025*. **E** EdU incorporation assay showing the percentage of EdU-positive cells in LMO4 overexpressing (LMO4-OE) and control (Con) OSCC cells. **F** Colony formation assay showing the number of colonies formed by LMO4-OE and Con in CAL27, HSC-2, and HSC-4 cell lines. **G** CCK-8 proliferation assay of LMO4-OE and Con in CAL27, HSC-2, and HSC-4 cell lines. **H** Transwell migration assay showing the invasive ability of LMO4-OE and Con in CAL27, HSC-2, and HSC-4 cell lines. **I** Western blot analysis showing the expression of epithelial-mesenchymal transition (EMT) markers (E-cadherin, N-cadherin, and Slug) in LMO4-OE and Con. GAPDH and β-actin were used as loading controls. *p < 0.05; **p < 0.01; ***p < 0.001.

We first examined LMO4 expression in 20 paired OSCC tumor and adjacent normal tissues by western blot, which revealed significantly elevated LMO4 protein levels in tumors, confirmed by densitometric analysis (Fig. 1A, B). Immunohistochemistry (IHC) further validated LMO4 upregulation, demonstrating strong nuclear and cytoplasmic staining in tumor cells, with minimal expression in adjacent normal tissues (Fig. 1C). Kaplan–Meier survival analysis showed that high LMO4 expression correlated with poorer overall survival (*p* = 0.025), underscoring its potential prognostic significance (Fig. 1D). We next assessed LMO4 levels in OSCC cell lines (SCC-9, HSC-4, HSC-2, CAL27) versus the non-tumorigenic epithelial line HaCat. Both RT-qPCR and western blot revealed markedly elevated LMO4 mRNA and protein levels in OSCC lines (Fig. S2A–C). To explore LMO4’s functional role, we established stable overexpression in CAL27, HSC-2, and HSC-4 cells, confirmed by western blot and RT-qPCR (Fig. S2D, S2E). Functionally, EdU incorporation assays revealed increased DNA synthesis in LMO4-overexpressing cells (Fig. 1E, Fig. S2F), while colony formation assays showed enhanced clonogenic potential (Fig. 1F). CCK-8 proliferation assays indicated that LMO4 sustained cell growth over 72 h (Fig. 1G). Wound healing and transwell assays demonstrated significantly enhanced migratory capacity upon LMO4 overexpression (Fig. 1H, Fig. S2G). Western blot analysis showed reduced E-cadherin and elevated N-cadherin and Slug expression in LMO4-overexpressing cells, suggesting EMT activation (Fig. 1I). Collectively, these findings indicate that LMO4 enhances OSCC malignancy by promoting proliferation and migration. We next explored the impact of LMO4 silencing.

### LMO4 knockdown impairs OSCC proliferation and motility

**Fig. 2:**
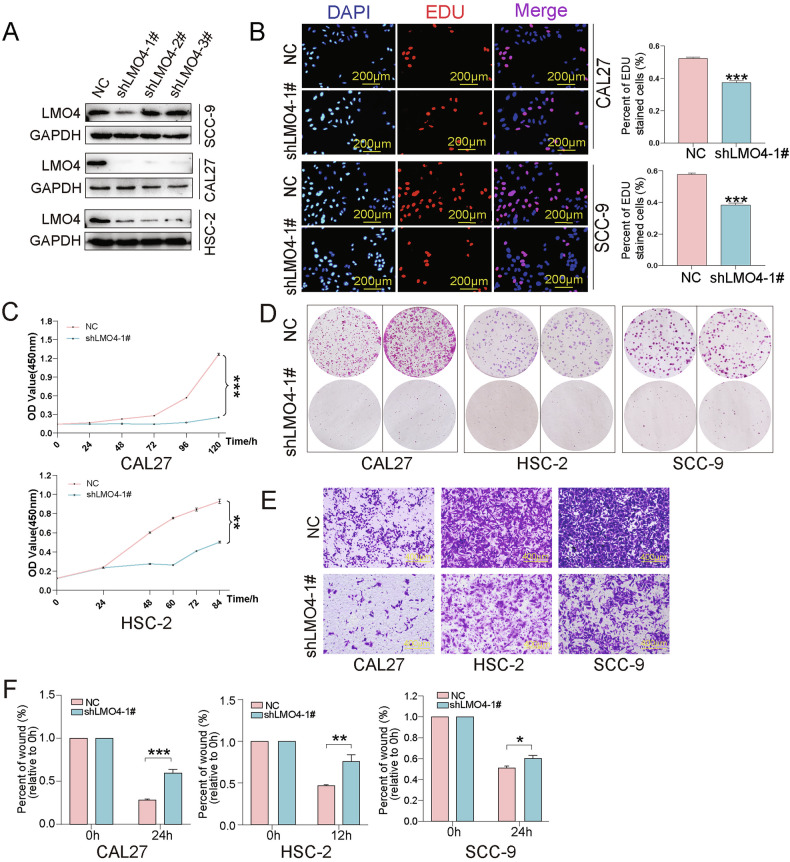
Knockdown of LMO4 inhibits OSCC cell proliferation, migration, and epithelial-mesenchymal transition (EMT). **A** Western blot analysis of LMO4 knockdown efficiency in OSCC cell lines using three different shRNA lentivirus. GAPDH and β-actin were used as loading controls. **B** EdU incorporation assay to evaluate cell proliferation in LMO4 knockdown (shLMO4) and negative control (NC) OSCC cells. **C** CCK-8 assay measuring cell growth over time in shLMO4 and NC OSCC cells. **D** Colony formation assay in shLMO4 and NC OSCC cells. **E** Transwell migration assay to assess cell migratory capacity in shLMO4 and NC OSCC cells. **F** Wound healing assay to analyze cell migration in shLMO4 and NC OSCC cells.

To assess the tumor-promoting function of LMO4, we silenced its expression using shRNA in CAL27, HSC-2, and SCC-9 cells. Western blotting confirmed effective knockdown in all lines (Fig. 2A). EdU assays showed that LMO4 knockdown significantly reduced DNA synthesis in CAL27 and SCC-9 cells (Fig. 2B). CCK-8 assays indicated suppressed proliferation following LMO4 depletion in CAL27 and HSC-2 (Fig. 2C), while colony formation assays showed diminished clonogenic potential across all three lines (Fig. 2D). To examine effects on motility, transwell assays demonstrated decreased migration in LMO4-deficient cells (Fig. 2E), supported by wound healing assays that revealed delayed closure compared with control cells (Fig. 2F). These data reinforce that LMO4 facilitates both proliferative and migratory capabilities in OSCC.

### LMO4 promotes RAB17 ubiquitin-mediated degradation

**Fig. 3:**
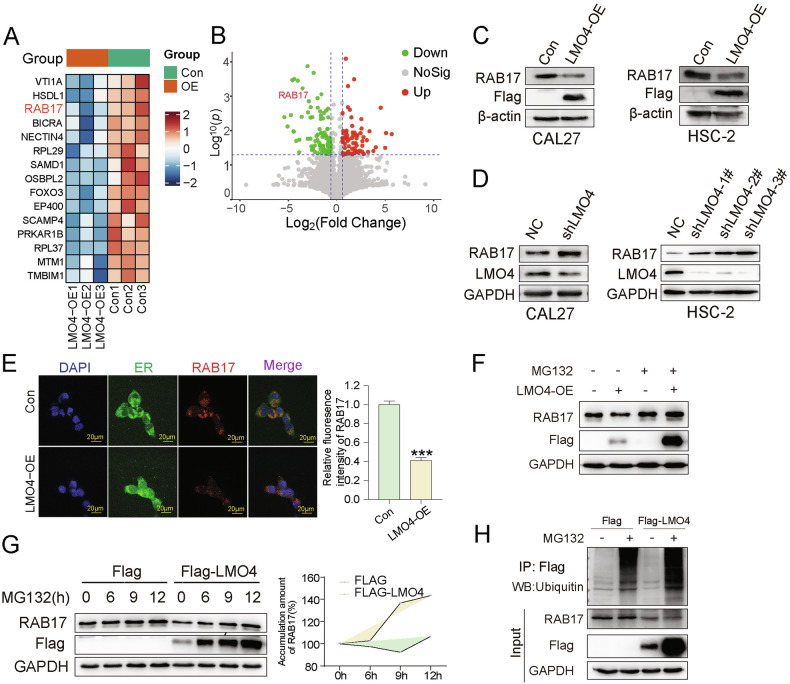
LMO4 regulates RAB17 expression *via* the ubiquitin-proteasome pathway. **A** Heatmap of selected upregulated and downregulated genes in LMO4-OE *vs*. Con cells. **B** Volcano plot representing the DEGs expression changes in LMO4-OE *vs*. Con cells. **C** Western blot analysis of RAB17 expression in LMO4-OE and Con cells. β-actin was used as a loading control. **D** Western blot analysis of RAB17 expression in shLMO4 and NC cells (NC). **E** Immunofluorescence images showing the localization and expression of RAB17 in CAL27 cells with LMO4-OE and Con groups. Endoplasmic reticulum (ER) staining is also shown. Scale bars: 20 μm. **F** Western blot analysis of RAB17 expression in CAL27 cells with LMO4-OE and Con groups, with or without MG132 treatment. **G** Time-course Western blot analysis of RAB17 degradation in LMO4-OE Cal27 cells treated with MG132 at different time points. The graph on the right quantifies the accumulation of RAB17 over time. **H** Co-immunoprecipitation analysis of LMO4 and RAB17 in the presence or absence of MG132 in Cal27. RAB17 ubiquitination was assessed after pulling down Flag-tagged LMO4.

To elucidate the mechanism by which LMO4 modulates OSCC progression, proteomic analysis was conducted in CAL27 cells overexpressing LMO4. Among the differentially expressed candidates, RAB17 emerged as significantly downregulated (Fig. 3A, B). We also examined RAB17 expression across multiple OSCC cell lines (Fig. S3A). Western blot analysis confirmed the suppression of RAB17 protein levels in LMO4-overexpressing CAL27 and HSC-2 cells (Fig. 3C), while LMO4 knockdown restored RAB17 protein expression (Fig. 3D). Notably, RT-qPCR showed no change in RAB17 transcript levels, indicating that LMO4 regulates RAB17 post-translationally (Fig. S3B–D). Immunofluorescence imaging revealed markedly reduced RAB17 expression in LMO4-overexpressing cells, predominantly localized to the endoplasmic reticulum, with quantification confirming a significant reduction in fluorescence intensity (Fig. 3E). To determine whether LMO4 promotes proteasome-mediated degradation of RAB17, we treated cells with MG132. Western blot analysis demonstrated that MG132 rescued RAB17 levels in LMO4-overexpressing cells (Fig. 3F), and time-course experiments showed progressive RAB17 accumulation with extended MG132 exposure (Fig. 3G). To further confirm that RAB17 is degraded *via* ubiquitination in response to LMO4, we performed co-immunoprecipitation assays. FLAG-tagged LMO4 co-immunoprecipitated with RAB17, and immunoblotting for ubiquitin revealed enhanced RAB17 ubiquitination in LMO4-overexpressing cells treated with MG132 (Fig. 3H). These results support the conclusion that LMO4 facilitates the degradation of RAB17 through a ubiquitin–proteasome-dependent pathway. However, the specific E3 ligase mediating this interaction remains to be identified in future studies.

### LMO4 promotes OSCC tumor growth and modulates RAB17 levels in vivo

**Fig. 4:**
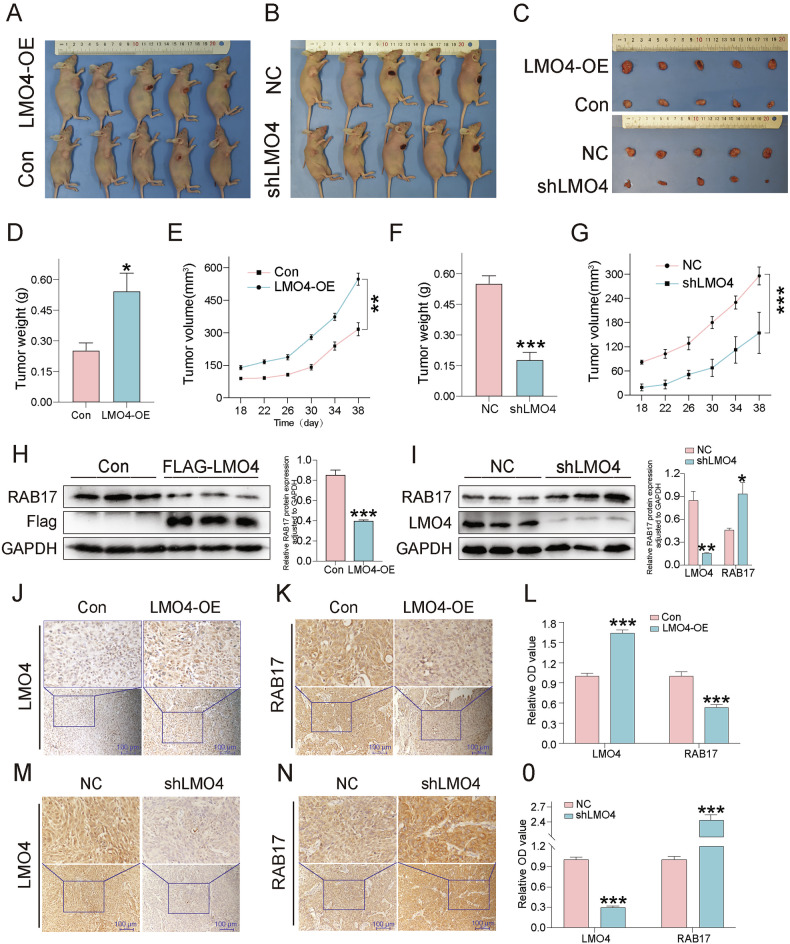
LMO4 promotes tumor growth and regulates RAB17 expression in vivo. **A** Representative image of mice with subcutaneous tumors derived from LMO4-OE and Con in CAL27 cells. **B** Representative images of mice with subcutaneous tumors derived from shLMO4 and NC in CAL27 cells. **C** Representative images of excised tumors from the different treatment groups: LMO4-OE, Con, shLMO4, and NC. **D** Tumor weight comparison between LMO4-OE and Con groups. **E** Tumor volume measurement over time for LMO4-OE and Con groups. **F** Tumor weight comparison between shLMO4 and NC groups. **G** Tumor volume measurement over time for shLMO4 and NC groups. **H** Western blot analysis of RAB17 expression in tumors derived from LMO4-OE and Con cells. GAPDH was used as a loading control. **I** Western blot analysis of RAB17 expression in tumors derived from shLMO4 and NC cells. GAPDH was used as a loading control. **J, K** Immunohistochemistry (IHC) staining of LMO4 and RAB17 in tumor sections from LMO4-OE and Con groups. **L** Quantification of IHC staining intensity for LMO4 and RAB17 in LMO4-OE and control groups. **M, N** IHC staining of LMO4 and RAB17 in tumor sections from shLMO4 and NC groups. **O** Quantification of IHC staining intensity for LMO4 and RAB17 in shLMO4 and NC groups.

To investigate the in vivo relevance of LMO4, we established subcutaneous xenograft models using CAL27 cells stably expressing LMO4 or shLMO4. Mice injected with LMO4-overexpressing cells developed significantly larger tumors with increased volume and weight compared to the control group (Fig. 4A, C–E). In contrast, tumors derived from LMO4-silenced cells were markedly smaller, with significantly reduced growth metrics relative to their respective controls (Fig. 4B, C, F, G). To validate RAB17 modulation by LMO4 in vivo, we analyzed tumor lysates by western blotting. Consistent with in vitro findings, LMO4-overexpressing tumors exhibited reduced RAB17 protein levels, whereas tumors from the shLMO4 group showed increased RAB17 expression (Fig. 4H–I). Immunohistochemistry further corroborated these results: LMO4-OE tumors displayed diminished RAB17 staining, while RAB17 expression was markedly elevated in tumors from the shLMO4 group (Fig. 4J, K, M, N). Quantitative analysis of staining intensities confirmed statistically significant differences across groups (Fig. 4L, O). Collectively, these in vivo data support a functional axis in which LMO4 drives OSCC progression by suppressing RAB17 expression.

### RAB17 regulates OSCC cell proliferation

**Fig. 5:**
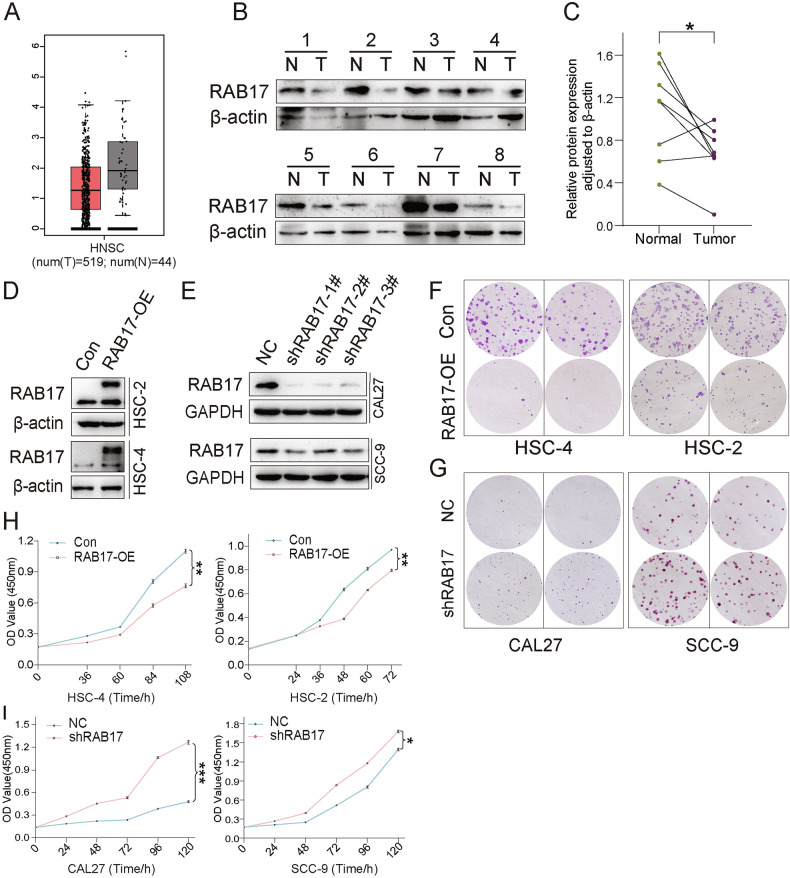
RAB17 expression and its role in OSCC cell proliferation and colony formation. **A** TCGA data analysis comparing RAB17 expression levels in normal (N) and tumor (T) tissues from head and neck squamous cell carcinoma (HNSC) patients. **B** Western blot analysis of RAB17 expression in paired normal (N) and tumor (T) tissues from OSCC patients, with β-actin as a loading control. **C** Quantification of RAB17 protein expression in normal and tumor tissues normalized to β-actin. **D, E** Western blot analysis of RAB17 expression in RAB17-OE and Con cells, as well as shRAB17 and NC cells, with GAPDH as a loading control. **F, G** Colony formation assay comparing the number of colonies formed by RAB17-OE and Con cells, as well as shRAB17 and NC cells. **H, I** CCK-8 assay measuring cell proliferation over time in RAB17-OE and Con cells, as well as shRAB17 and NC cells.

To clarify the functional role of RAB17 in OSCC, we first examined its expression profile. TCGA analysis revealed that RAB17 was significantly upregulated in head and neck squamous cell carcinoma (HNSC) tissues compared to normal samples (Fig. 5A). Western blot analysis of paired OSCC patient samples further confirmed elevated RAB17 protein levels in tumors relative to adjacent normal tissues (Fig. 5B), with densitometric quantification demonstrating statistical significance (Fig. 5C). We next manipulated RAB17 expressions in OSCC cell lines. Stable RAB17 overexpression (RAB17-OE) in HSC-4 and HSC-2 cells and knockdown (shRAB17) in CAL27 and SCC-9 cells were confirmed by western blot (Fig. 5D, E). Colony formation assays revealed that RAB17-OE enhanced clonogenicity, whereas RAB17 silencing significantly suppressed colony formation (Fig. 5F, G). Consistently, CCK-8 proliferation assays showed accelerated growth in RAB17-OE cells and diminished proliferation in shRAB17 cells over time (Fig. 5H, I). These findings suggest that RAB17 positively regulates OSCC cell proliferation and tumorigenic potential.

### RAB17 suppresses OSCC cell motility and epithelial-mesenchymal transition

**Fig. 6:**
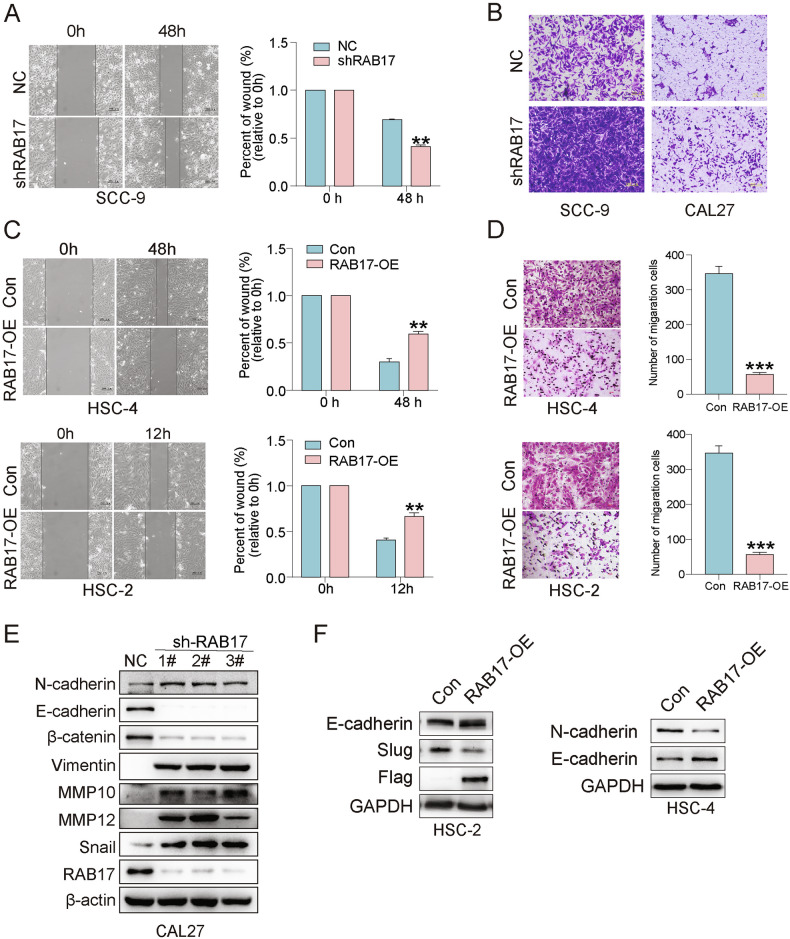
RAB17 regulates OSCC cell migration, invasion, and epithelial-mesenchymal transition (EMT). **A** Wound healing assay comparing the migration of shRAB17 and NC cells in SCC-9. Quantification of wound closure is shown on the right. **B** Transwell migration assay comparing the migration of shRAB17 and NC cells in OSCC cell lines. **C** Wound healing assay comparing the migration of RAB17-OE and Con cells in OSCC cell lines at 12 h and 48 h time points. Quantification of wound closure is shown on the right. **D** Transwell migration assay comparing the invasion of RAB17-OE and Con cells. Quantification of migrated cells is shown on the right. **E** Western blot analysis of EMT-related markers (N-cadherin, E-cadherin, β-catenin, Vimentin, MMP10, MMP12, and Snail) in shRAB17 and NC cells. β-actin was used as a loading control. **F** Western blot analysis of EMT markers (E-cadherin, N-cadherin, Slug, and Flag) in RAB17-OE and Con cells.

To explore whether RAB17 affects OSCC invasiveness, we evaluated cell migration using wound healing and transwell assays. Knockdown of RAB17 in SCC-9 and CAL27 cells resulted in markedly impaired wound closure and reduced transwell migration compared to control cells (Fig. 6A, B). Conversely, overexpression of RAB17 in HSC-2 and HSC-4 cells significantly enhanced migratory capacity (Fig. 6C, D). Western blot analysis of EMT-associated markers demonstrated that RAB17 knockdown upregulated mesenchymal markers (N-cadherin, Vimentin, Snail) and matrix metalloproteinases (MMPs, MMP10 and MMP12), while downregulating epithelial markers (E-cadherin, β-catenin) (Fig. 6E). In contrast, RAB17 overexpression led to reduced epithelial marker expression and increased N-cadherin and Slug (Fig. 6F). Together, these results indicate that RAB17 suppresses EMT and invasive behavior in OSCC cells, further supporting its tumor-suppressive role.

### LMO4 and RAB17 modulate ferroptosis *via* the Nrf2-Keap1 axis

**Fig. 7:**
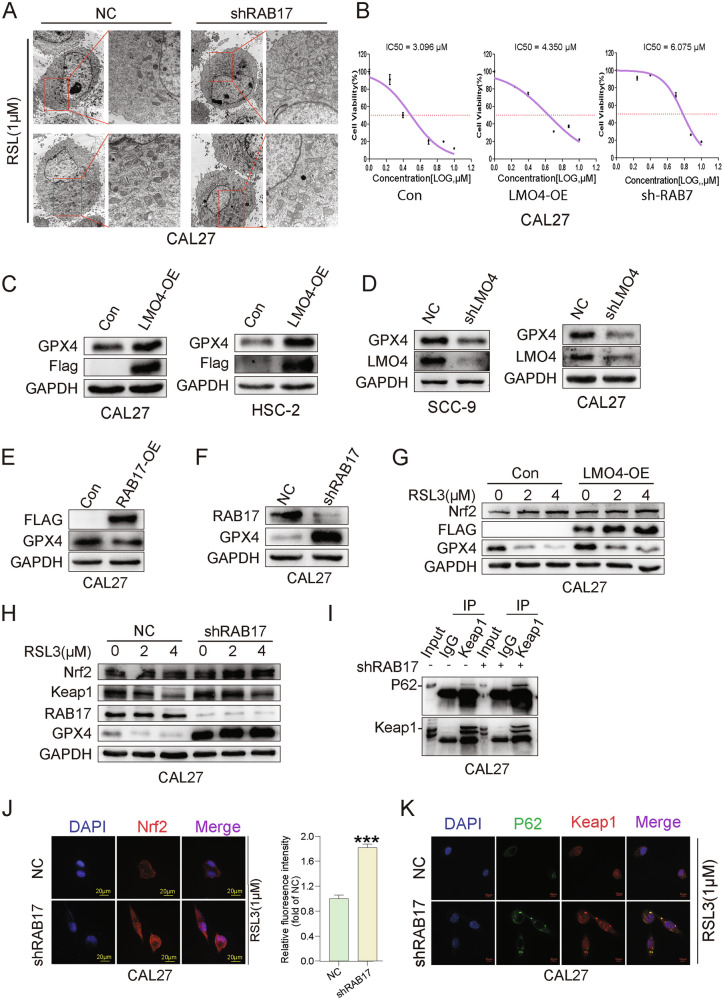
RAB17 and LMO4 regulate ferroptosis and the Nrf2-Keap1 signaling pathway in OSCC cells. **A** Transmission electron microscopy image showing the ultrastructure of mitochondria in CAL27 cells treated with shRAB17 or NC group under RSL3 (a ferroptosis inducer) treatment. **B** Dose-response curves showing cell viability in CAL27 cells with LMO4-OE, shRAB17, and Con cells treated with RSL3. IC50 values are indicated. **C** Western blot analysis of GPX4 expression in LMO4-OE and Con cells, with GAPDH used as a loading control. **D** Western blot analysis of GPX4 and LMO4 expression in shLMO4 and NC cells, with GAPDH used as a loading control. **E** Western blot analysis of GPX4 expression in RAB17-OE and Con cells. **F** Western blot analysis of GPX4 expression in shRAB17 and NC cells. **G** Western blot analysis of Nrf2, FLAG, GPX4, and GAPDH in LMO4-OE and Con cells treated with RSL3. **H** Western blot analysis of Nrf2, Keap1, RAB17, and GPX4 expression in shRAB17 and NC cells treated with RSL3. **I** Co-immunoprecipitation analysis of P62 and Keap1 interaction in shRAB17 and NC cells. **J** Immunofluorescence images showing Nrf2 nuclear localization in shRAB17 and NC cells under RSL3 treatment, with quantification of fluorescence intensity on the right. **K** Immunofluorescence images showing P62 and Keap1 colocalization in shRAB17 and NC cells under RSL3 treatment.

To examine the impact of LMO4 and RAB17 on ferroptosis, we treated CAL27 cells with the GPX4 inhibitor RSL3. Transmission electron microscopy revealed hallmark mitochondrial abnormalities indicative of ferroptosis-such as cristae reduction and outer membrane rupture-in shRAB17 cells upon RSL3 exposure (Fig. 7A). Dose–response analysis showed that both LMO4 overexpression and RAB17 knockdown increased RSL3 IC50 values, suggesting reduced ferroptotic sensitivity (Fig. 7B).

Western blotting confirmed that LMO4 overexpression elevated GPX4 protein levels in CAL27 and HSC-2 cells, whereas LMO4 knockdown reduced GPX4 in SCC-9 and CAL27 cells (Fig. 7C, D). Similarly, RAB17 overexpression increased GPX4 expression, while knockdown diminished its levels (Fig. 7E, F). Further RSL3 dose-dependent experiments revealed that GPX4 was downregulated in both LMO4-OE and control groups but remained relatively preserved in shRAB17 cells, consistent with resistance to ferroptosis (Fig. 7G). Notably, nuclear accumulation of NRF2 was enhanced in shRAB17 cells upon RSL3 treatment, implicating Nrf2-Keap1 dysregulation (Fig. 7H). Co-immunoprecipitation assays demonstrated increased p62-Keap1 interaction in shRAB17 cells (Fig. 7I), suggesting that p62 sequesters Keap1 and stabilizes NRF2. Confocal microscopy confirmed nuclear NRF2 accumulation and increased colocalization of p62 and Keap1 in shRAB17 cells compared to controls (Fig. 7J, K). These data indicate that LMO4 and RAB17 regulate ferroptosis sensitivity in OSCC through modulation of the Nrf2-Keap1-GPX4 axis.

## Discussion

Oral squamous cell carcinoma (OSCC) remains a clinically challenging malignancy due to its high metastatic potential and limited therapeutic targets [24]. Despite increasing insights into the molecular mechanisms of OSCC progression, clinically actionable biomarkers are scarce. Our findings position LMO4 as a critical oncogenic factor in OSCC, supported by its elevated expression in tumor tissues and correlation with poor patient prognosis [25]. Consistent with prior reports implicating LMO4 in breast, gastric, and pancreatic cancers, we demonstrate that LMO4 enhances OSCC cell proliferation, migration, and in vivo tumor growth [26, 27].

To uncover downstream mediators of LMO4, we performed proteomic profiling in LMO4-overexpressing OSCC cells, identifying RAB17 as a significantly downregulated protein. While LMO4 has classically been described as a transcriptional co-regulator, our data suggests that it exerts post-translational control over RAB17 *via* proteasome-mediated degradation. Notably, LMO4 overexpression promoted RAB17 ubiquitination without altering its mRNA levels, and the inhibition of the proteasome restored RAB17 protein abundance. These results uncover a novel regulatory mechanism in which LMO4 destabilizes RAB17 through the ubiquitin-proteasome system. Among the differentially expressed proteins identified by mass spectrometry, RAB17 was prioritized for further investigation due to its epithelial-specific expression profile, involvement in membrane trafficking and recycling, and previously reported tumor-suppressive functions in other epithelial cancers. Moreover, its substantial downregulation and distinct cellular functions aligned with key phenotypic changes observed in LMO4-overexpressing cells, thereby providing a compelling rationale for focusing on RAB17 as a novel downstream effector of LMO4 in OSCC.

We also explored whether the LMO4-RAB17 axis affects noncanonical cancer-related pathways. Recent studies have highlighted the therapeutic potential of ferroptosis induction in treatment-resistant cancers [28]. Our findings reveal that LMO4 confers ferroptosis resistance in OSCC by upregulating GPX4, a lipid hydroperoxidase critical for ferroptosis inhibition [29, 30]. Moreover, RAB17 silence increased nuclear NRF2 levels and GPX4 expression *via* enhanced p62-Keap1 interaction, suggesting that RAB17 modulates oxidative stress responses [31]. These results suggest that RAB17 regulates redox homeostasis in OSCC through modulating the NRF2-GPX4 axis, thereby influencing ferroptosis sensitivity.

Taken together, our data identify LMO4 as a dual-function oncogenic driver in OSCC, facilitating both RAB17 degradation and ferroptosis resistance. Targeting the LMO4-RAB17-NRF2-GPX4 axis may offer a novel therapeutic strategy for sensitizing OSCC cells to ferroptosis inducers and improving outcomes in patients with aggressive disease.

## Conclusion

This study identifies LMO4 as a key oncogenic driver in OSCC through its regulation of RAB17 and ferroptosis resistance. We demonstrate that LMO4 is significantly upregulated in OSCC and associated with poor patient prognosis. Mechanistically, LMO4 promotes tumor cell proliferation and migration by downregulating RAB17 *via* ubiquitin-mediated proteasomal degradation. RAB17 functions as a tumor suppressor, and its loss contributes to malignant phenotypes and elevated ferroptosis resistance *via* the NRF2-GPX4 axis. These findings establish the LMO4-RAB17 regulatory axis as a critical pathway in OSCC progression and suggest that targeting this axis may offer novel therapeutic opportunities for patients with advanced diseases. Further studies are warranted to explore its potential role in mediating chemoresistance and clinical outcomes.

## Supplementary information

SUPPLEMENTAL MATERIAL

Original Western blot images were cropped for presentation

## Data Availability

All data generated or analyzed during this study are included in this published article (and its supplementary information files).
