# Supplementary material for: LMO4 promotes OSCC progression by inducing RAB17 degradation and ferroptosis resistance
Source: Cell Death Dis. 2025 Nov 10;16(1):820. doi: 10.1038/s41419-025-08171-1 (PMC12602706; doi:10.1038/s41419-025-08171-1)
Supplement: Supplementary file 1 — SUPPLEMENTAL MATERIAL [file 41419_2025_8171_MOESM1_ESM.docx]

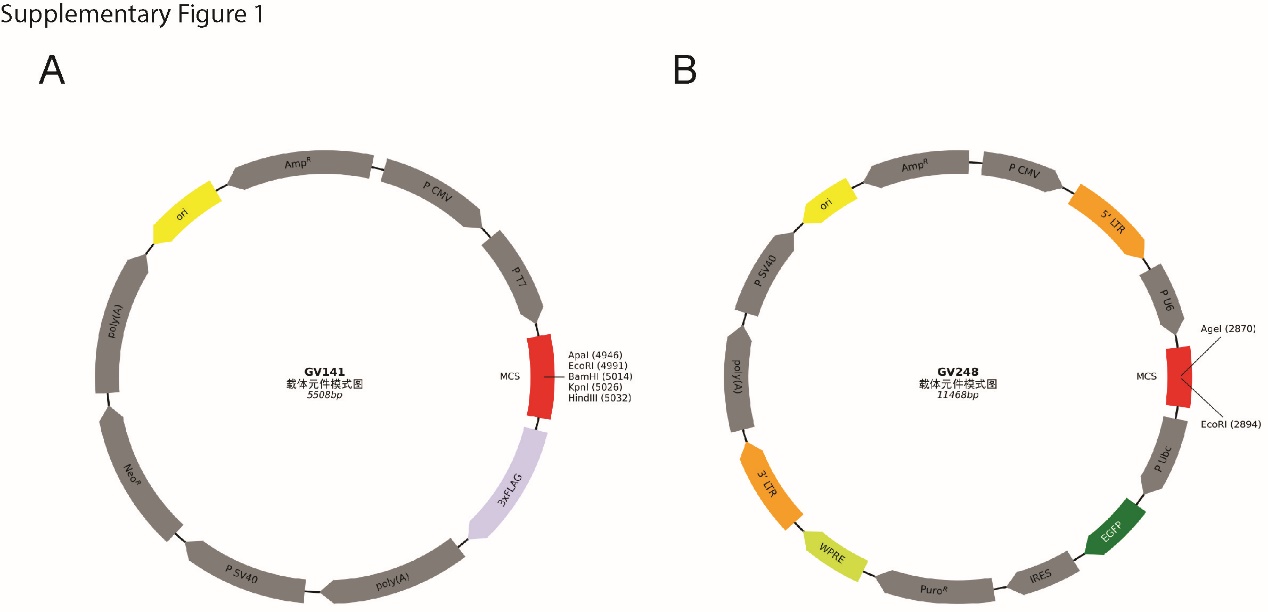


**Supplementary Figure 1. Schematic maps of plasmid constructs.**

(A) Map of the GV141 plasmid (5508 bp) showing key features, including the origin of replication (ori, yellow), antibiotic resistance genes (Amp^R^ and Neo^R^, gray), promoters (P CMV and P T7, gray), polyadenylation signals (poly(A), gray), 3×FLAG tag (light purple), and multiple cloning site (MCS, red) with indicated restriction enzyme sites (ApaI 4946, EcoRI 4991, BamHI 5014, KpnI 5026, HindIII 5032). (B) Map of the GV248 plasmid (11,468 bp) showing key features, including the origin of replication (ori, yellow), promoters (P CMV, P U6, P SV40, gray), antibiotic resistance gene (Puro^R^, gray), polyadenylation signal (poly(A), gray), long terminal repeats (5′ LTR and 3′ LTR, orange), WPRE (yellow-green), IRES (gray), EGFP reporter (green), and multiple cloning site (MCS, red) with indicated restriction enzyme sites (AgeI 2870, EcoRI 2894).


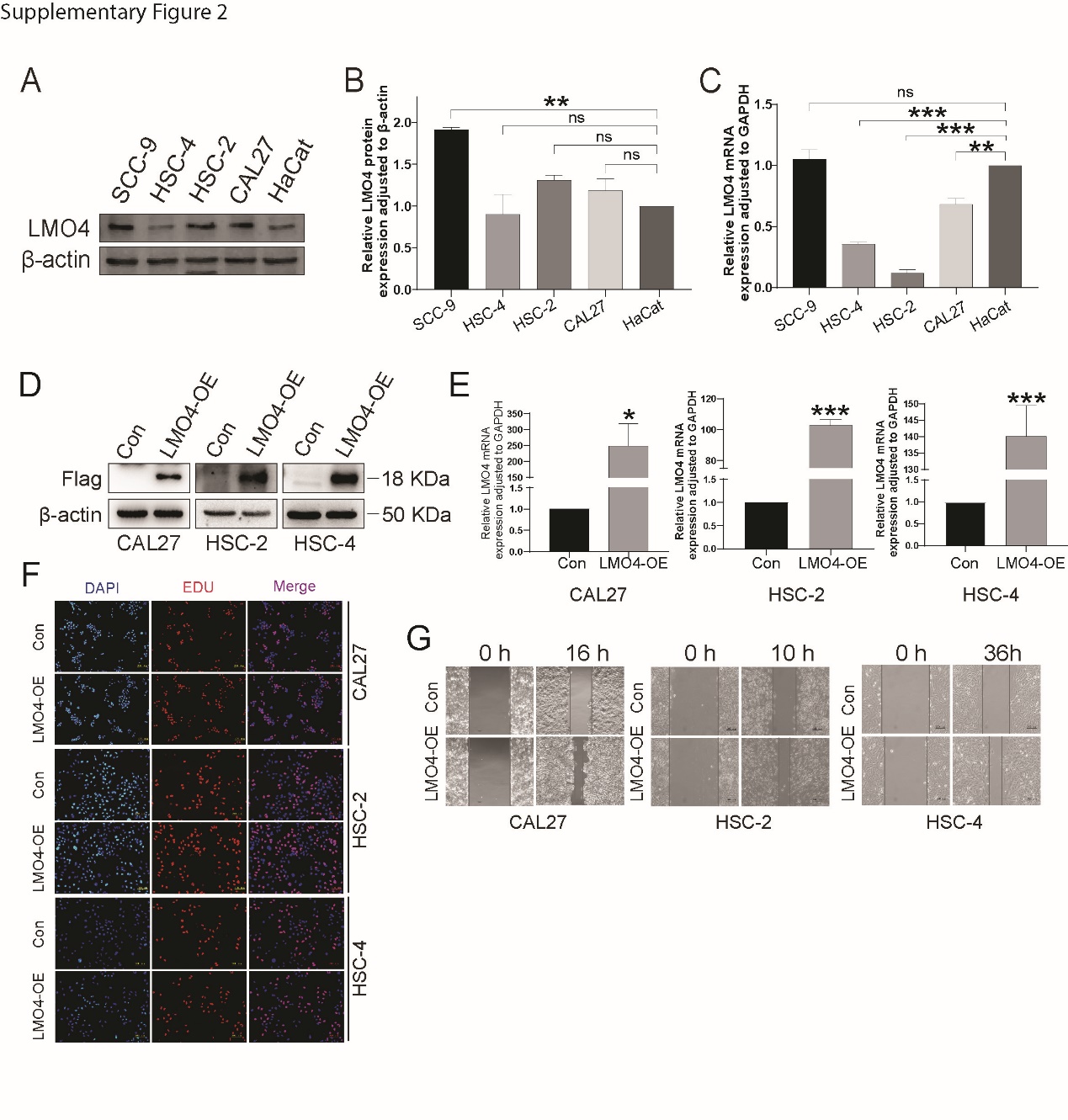


**Supplementary Figure 2. LMO4 expression and functional assays in OSCC cell lines.**

(A) Western blot analysis of LMO4 expression in various OSCC cell linesand the HaCat cell line. β-actin was used as a loading control. (B) Quantification of LMO4 protein expression normalized to β-actin in OSCC and HaCat cell lines. (C) Relative mRNA expression levels of LMO4 in OSCC cell lines and HaCat, normalized to GAPDH. (D) Western blot analysis of Flag-tagged LMO4 overexpression (LMO4-OE) in OSCC cells. β-actin was used as a loading control. (E) Quantification of LMO4 mRNA and protein levels in CAL27, HSC-2, and HSC-4 cells with LMO4-OE compared to Con. (F) EdU incorporation assay showing proliferating cells (EDU-positive) in LMO4-OE and Con cells. DAPI was used for nuclear staining. (G) Wound healing assay showing the migration ability of LMO4-OE and Con cells at indicated time points.


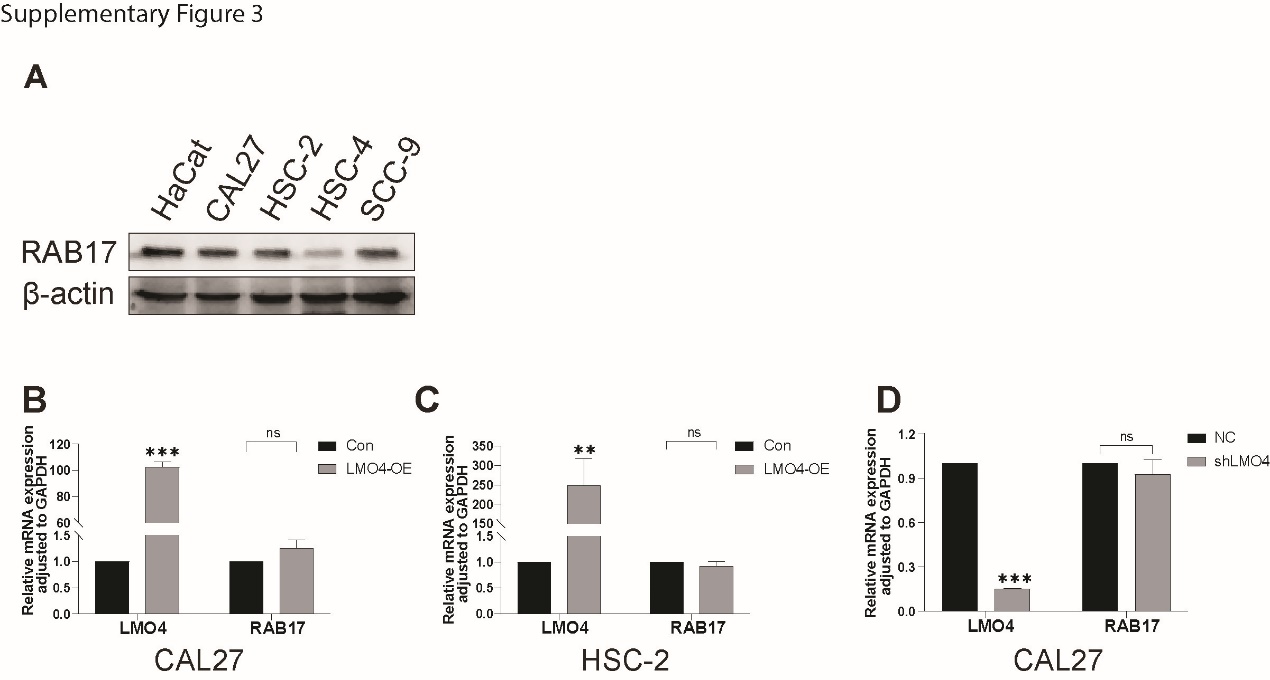


**Supplementary Figure 3. RAB17 expression and its relationship with LMO4 in OSCC cell lines.**

(A) Western blot analysis of RAB17 protein expression in OSCC cell lines (CAL27, HSC-2, HSC-4, SCC-9) and the HaCat cell line. β-actin was used as a loading control. (B) RT-qPCR analysis of LMO4 and RAB17 mRNA expression levels in CAL27 cells with LMO4 overexpression (LMO4-OE) and control (Con). Data were normalized to GAPDH. (C) RT-qPCR analysis of LMO4 and RAB17 mRNA expression levels in HSC-2 cells with LMO4-OE and control. Data were normalized to GAPDH. (D) RT-qPCR analysis of LMO4 and RAB17 mRNA expression levels in CAL27 cells with LMO4 knockdown (shLMO4) and negative control (NC). Data were normalized to GAPDH.

**Supplementary Table S1. List of Primary Antibodies and Suppliers**

| **Antibody** | **Supplier** | **Catalog Number** |
| --- | --- | --- |
| LMO4 | Abcam | ab23696 |
| RAB17 | Abcam | ab203625 |
| GAPDH | Proteintech | 60004-1-Ig |
| E-cadherin | CST | #3195 |
| N-cadherin | CST | #4061 |
| Slug | CST | #9585 |
| Snail | CST | #3879 |
| Vimentin | CST | #5741 |
| MMP10 | Santa Cruz Biotechnology | sc-80197 |
| MMP12 | Abcam | ab52897 |
| Ubiquitin | CST | #3936 |
| GPX4 | Proteintech | 67763-1-PBS |
| Nrf2 | Proteintech | 16396-1-AP |
| Keap1 | Proteintech | 10503-2-AP |
| P62/SQSTM1 | CST | #5114 |
| Flag-tag | CST | #14793 |
| β-actin | Abcam | ab8227 |

**Supplementary Table S2. Primer sequences used for quantitative real-time PCR**

| **Gene** | **Gene ID** |  | **Primer (5′–3′)** | **Product Size (bp)** |
| --- | --- | --- | --- | --- |
| **LMO4** | **8543** | **Forward** | **TGCAGATGAGGAGGAGGAGA** | **198** |
|  |  | **Reverse** | **CTTGGTGCTGCTGTTGTTGT** |  |
| **RAB17** | **64284** | **Forward** | **AGGAGGAGGAGGAGGAGGAG** | **180** |
|  |  | **Reverse** | **TGGGAGGAGGAGGAGGAGGA** |  |
| **GAPDH** | **2597** | **Forward** | **GTCTCCTCTGACTTCAACAGCG** | **177** |
|  |  | **Reverse** | **ACCACCCTGTTGCTGTAGCCAA** |  |
