# Supplementary material for: LMO4 promotes OSCC progression by inducing RAB17 degradation and ferroptosis resistance
Source: Cell Death Dis. 2025 Nov 10;16(1):820. doi: 10.1038/s41419-025-08171-1 (PMC12602706; doi:10.1038/s41419-025-08171-1)

Figure 1A

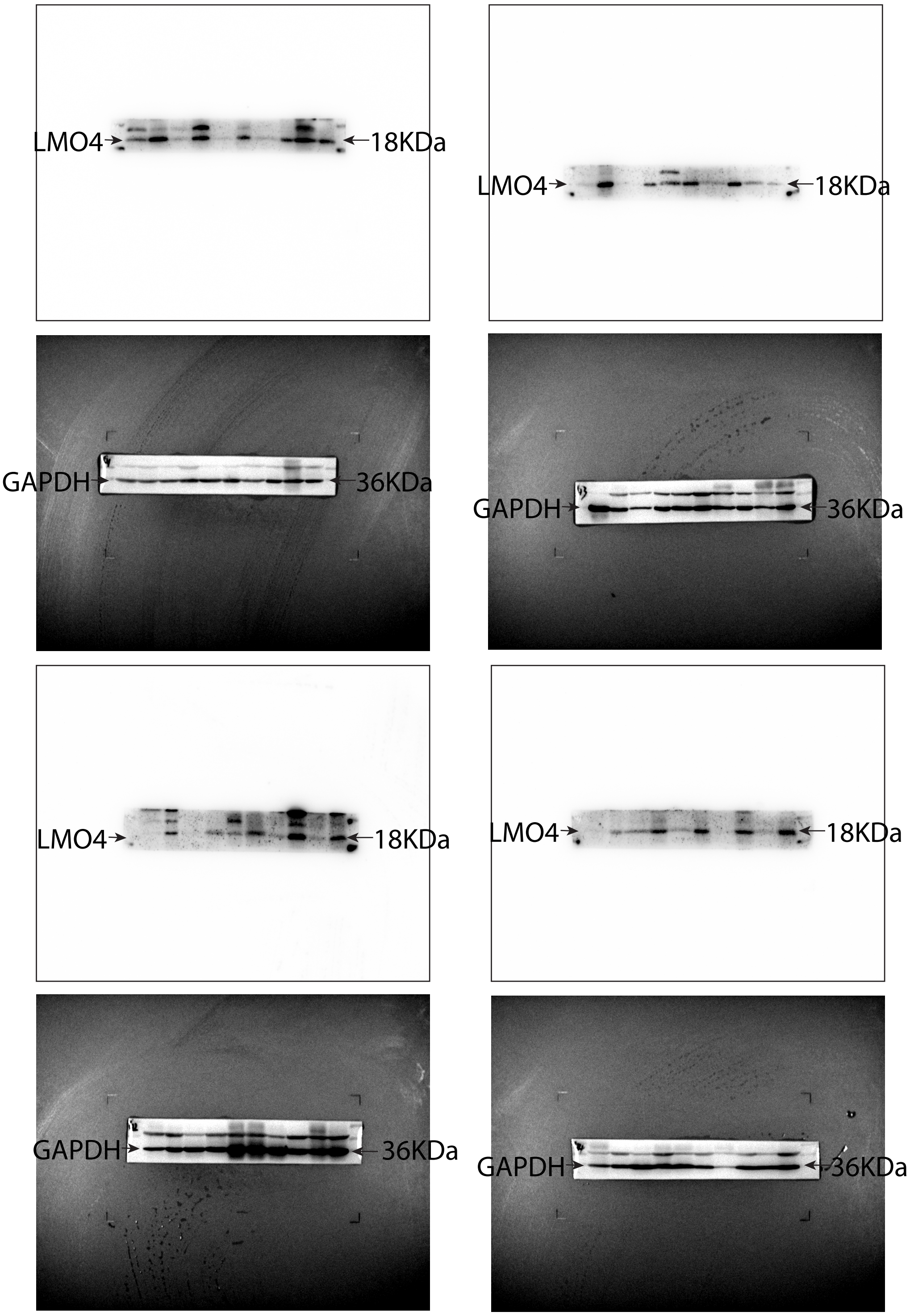
LMO4
18KDa
LMO4
18KDa
GAPDH
36KDa
GAPDH
36KDa
LMO4
18KDa
LMO4
18KDa
GAPDH
36KDa
GAPDH
36KDa

Figure 1I-1

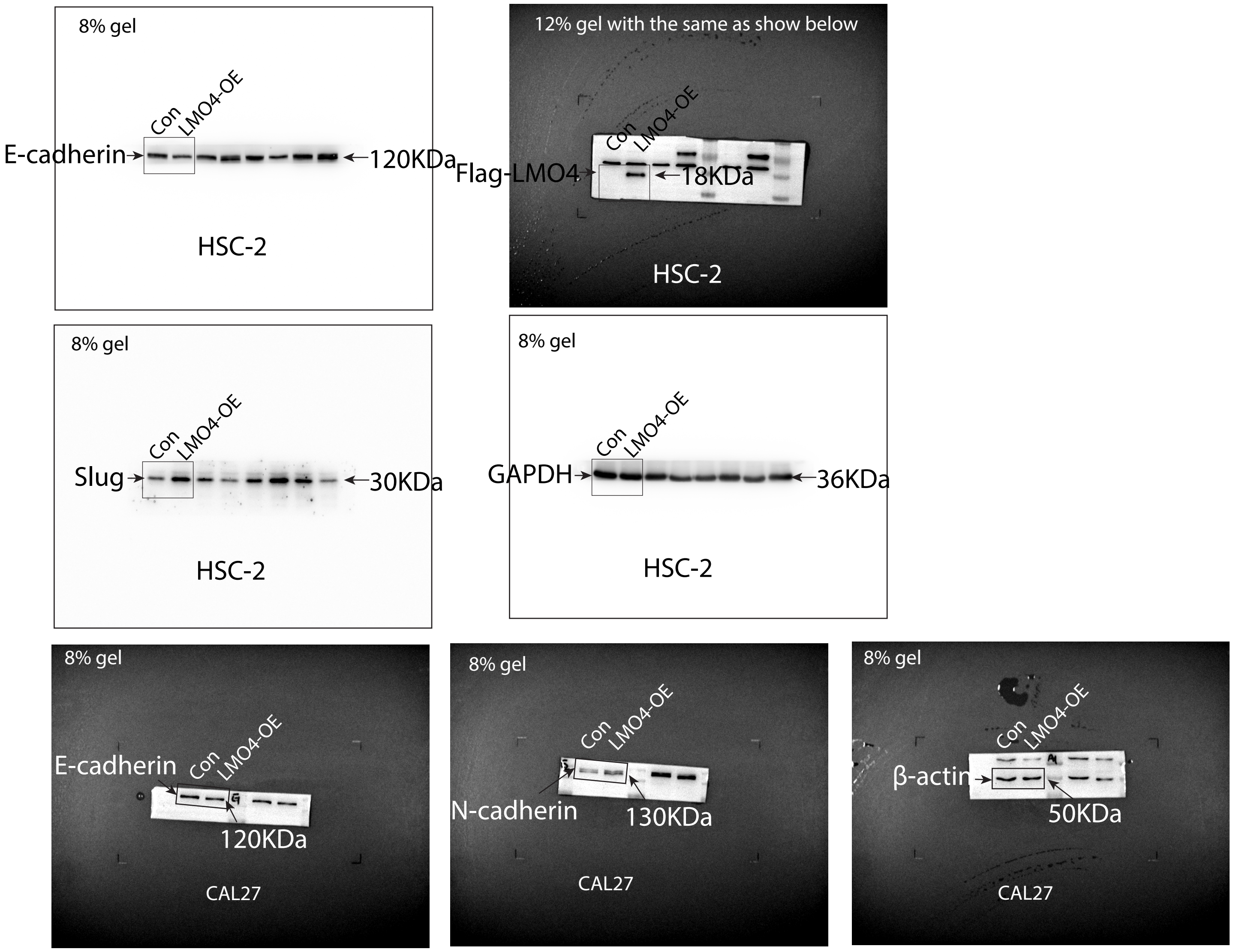

Figure 1I-2

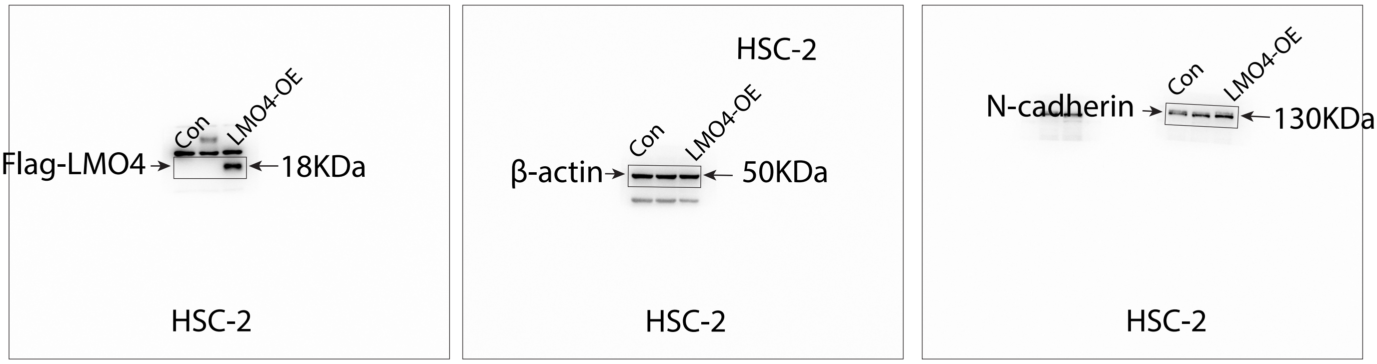

Figure 2A-1

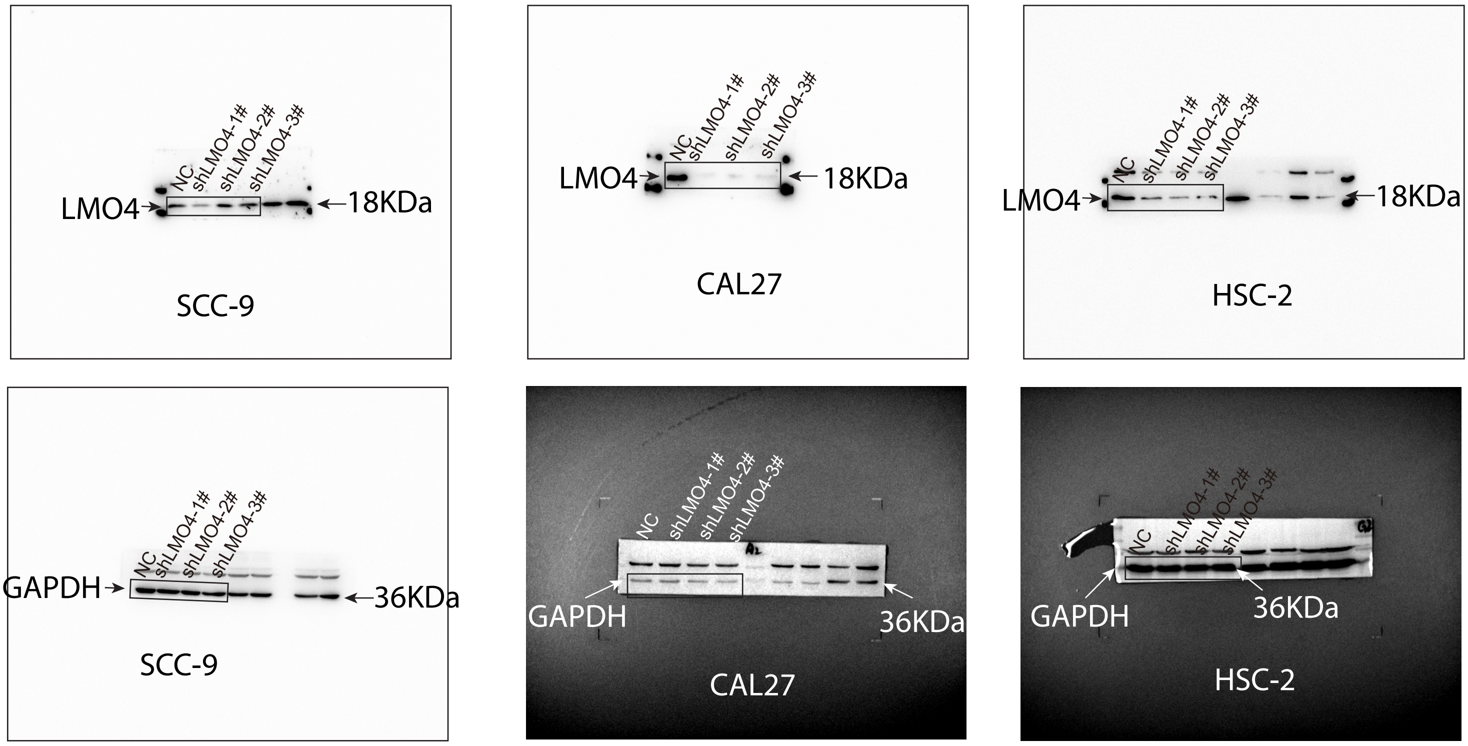

Figure 2A-2

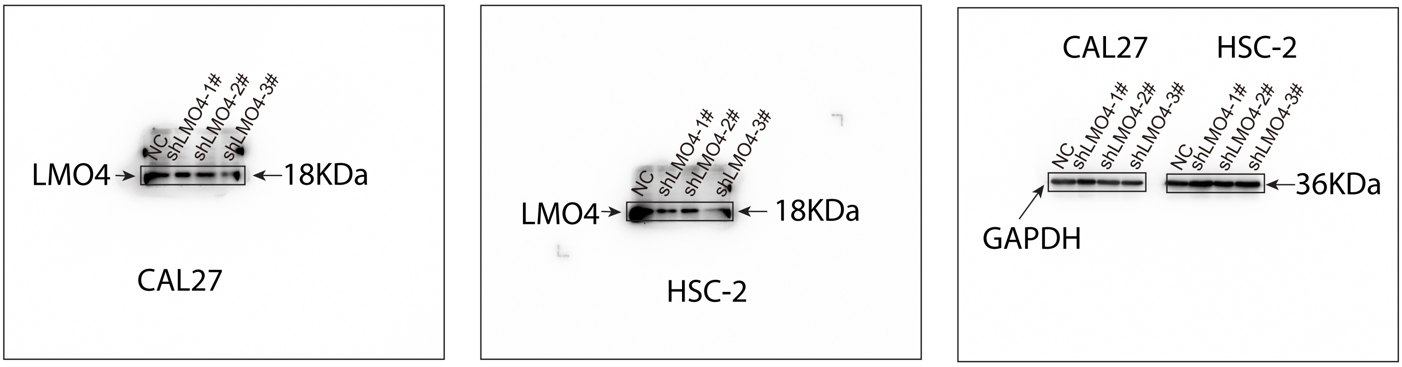

Figure 2A-3

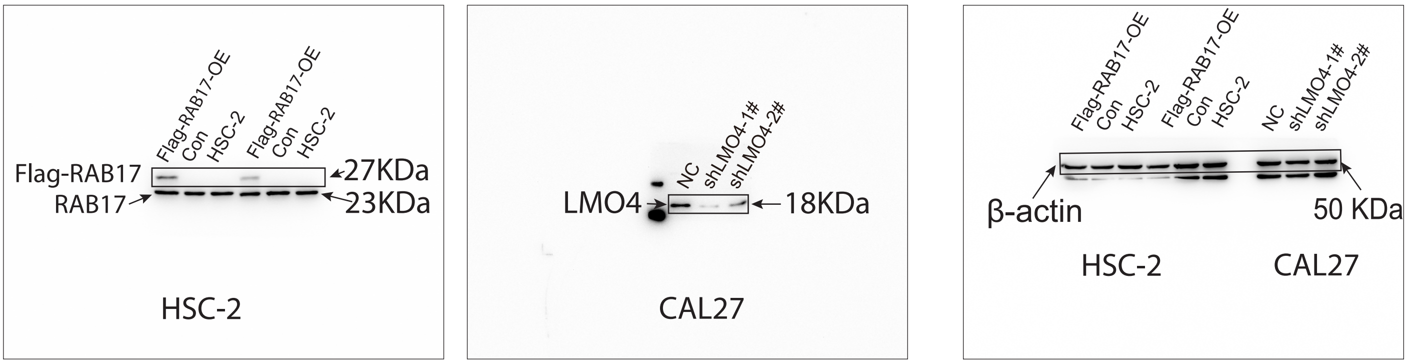

Detection of RAB17 overexpression efficiency in HSC-2 cells and LMO4 knockdown efficiency in Cal27/HSC-2 cells

Figure 2A-4

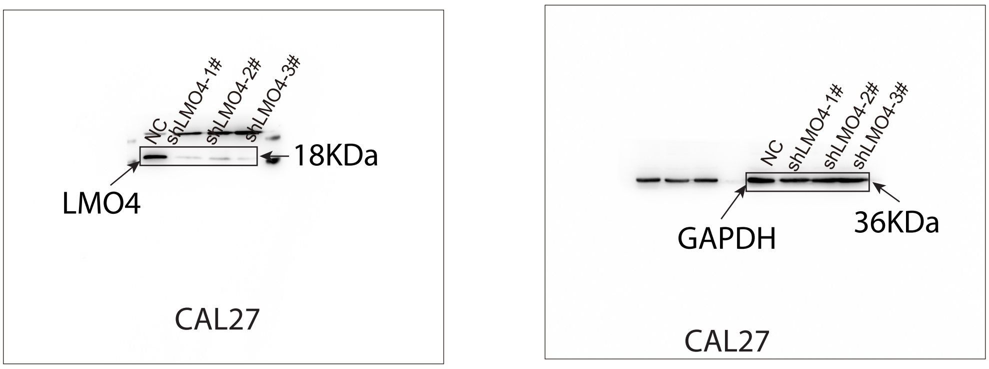

20230324

Figure 3D-left-1

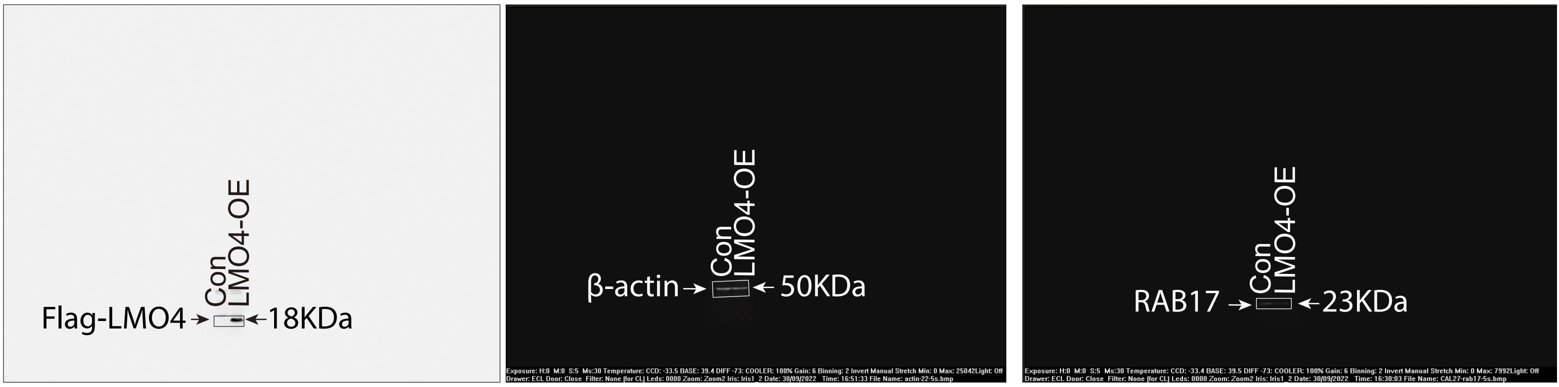

CAL27

Figure 3D-left-2,3

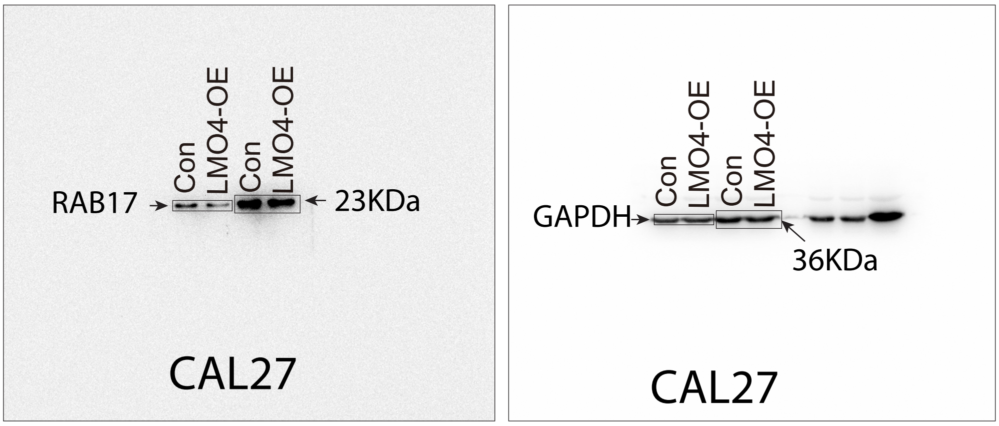

We did not detect flag-lmo4 expression

Figure 3D-right-1

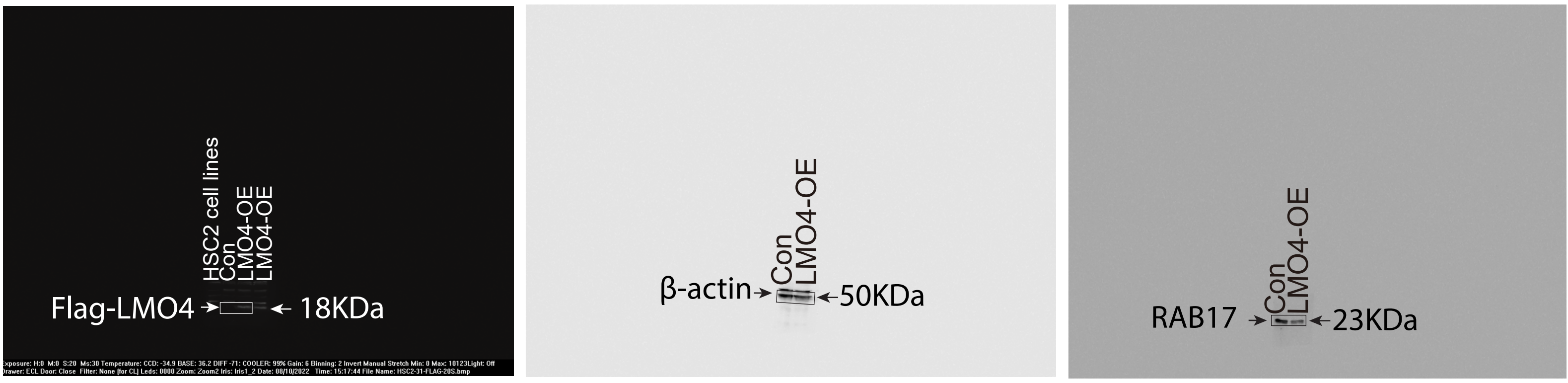

HSC-2

Figure 3D-left or right 3

We did not detect flag-lmo4 expression

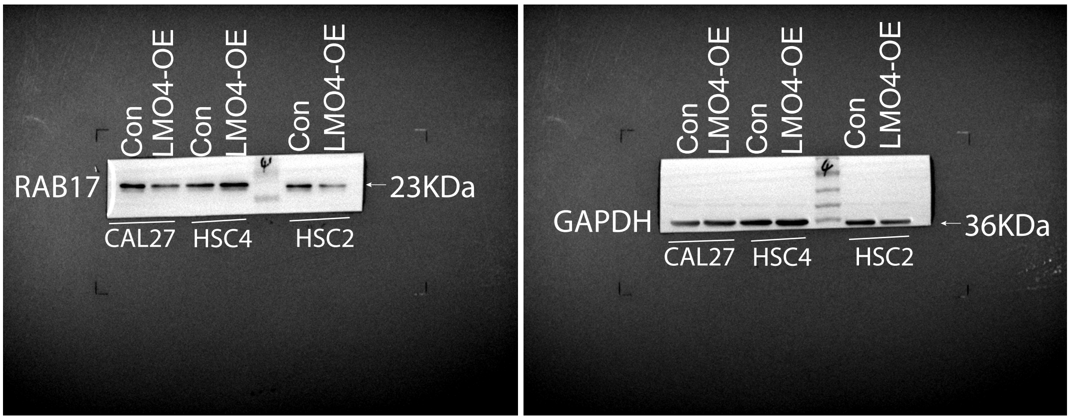

Figure 3G

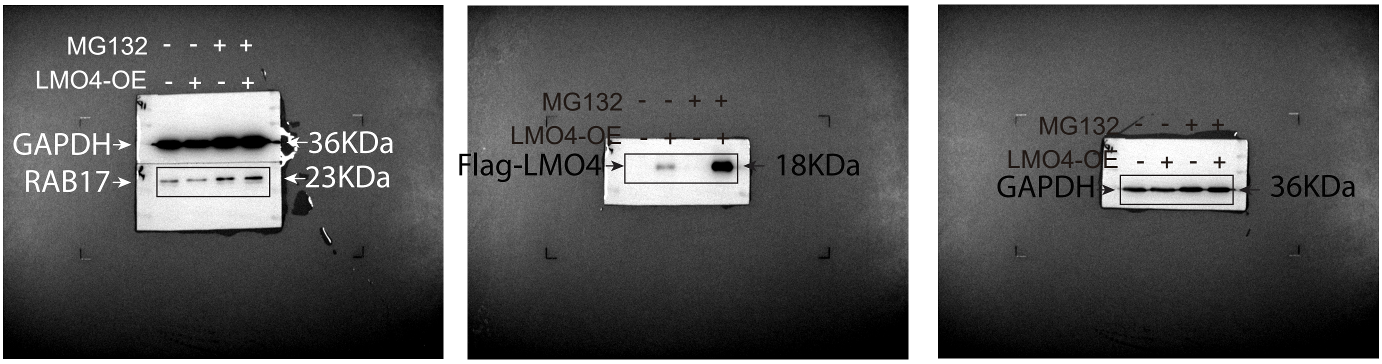

Figure 3H

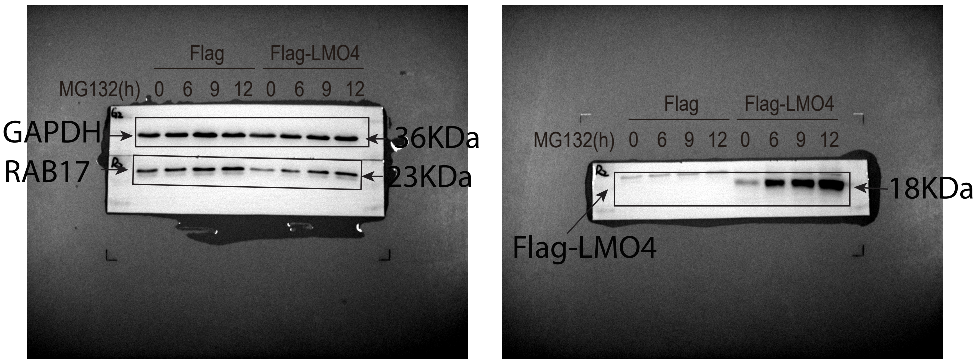

Figure 3I

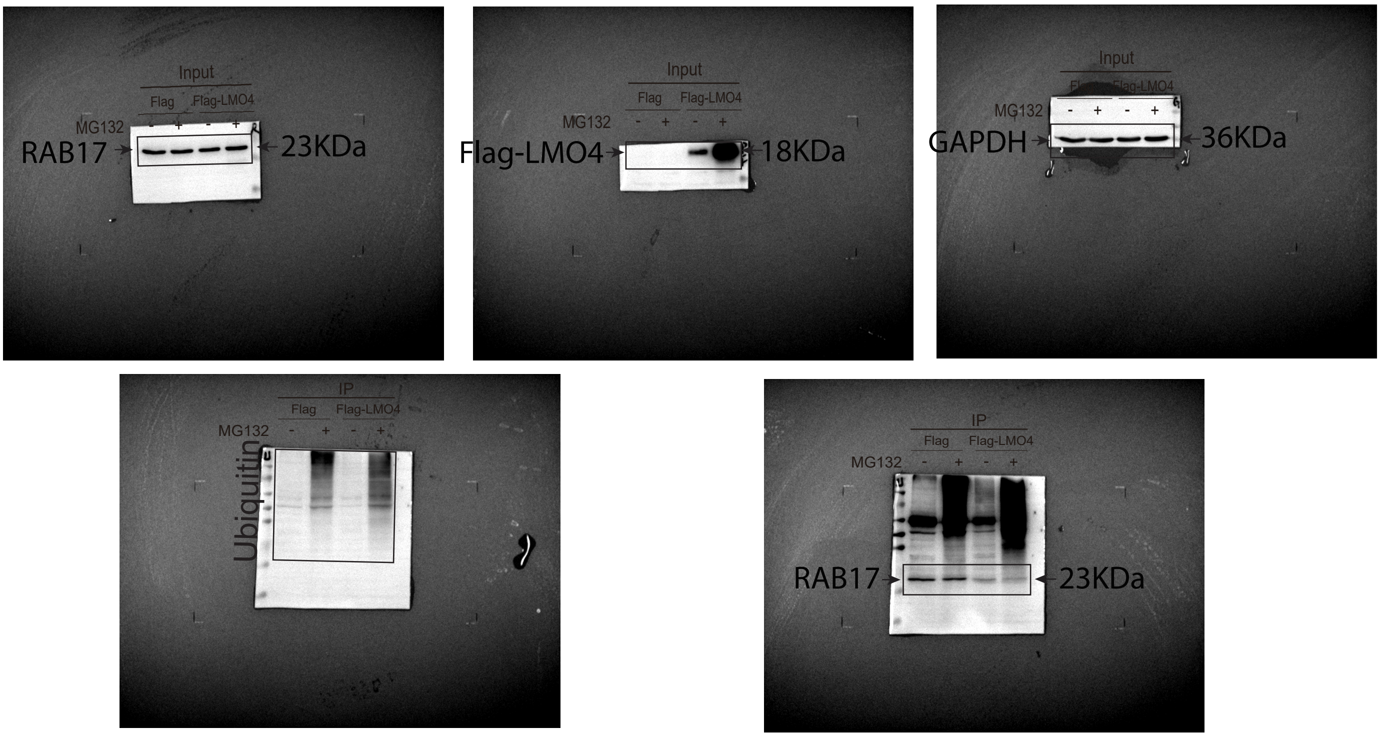

Figure 4H

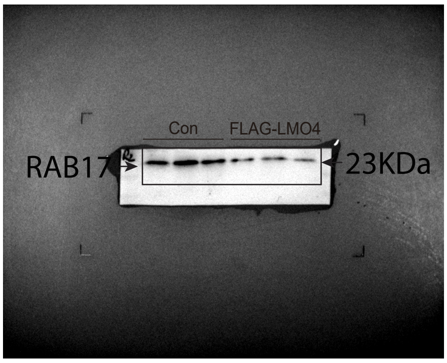

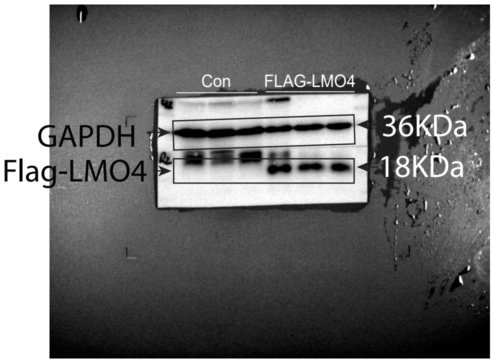

Figure 4I

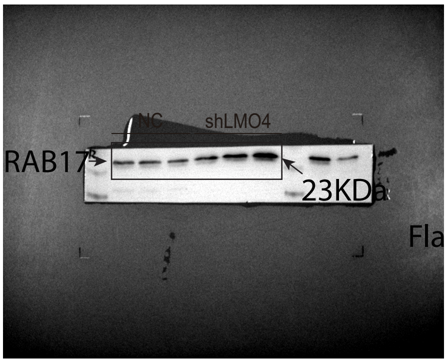

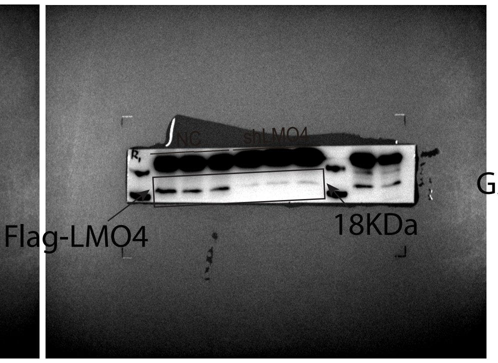

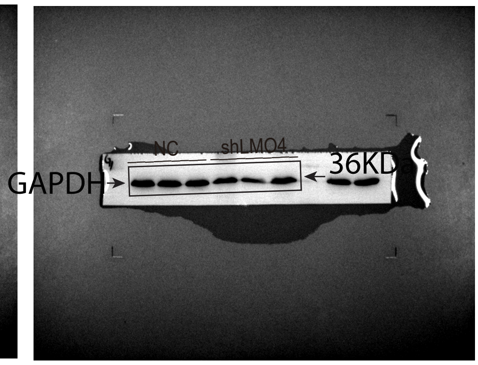

Figure 5B

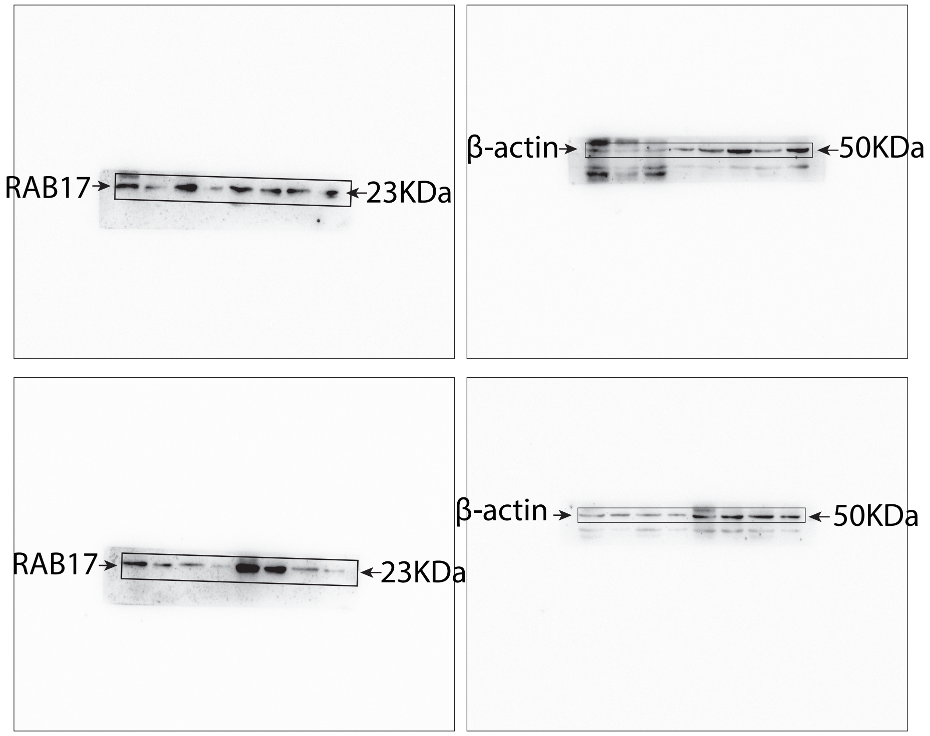

Figure 5D

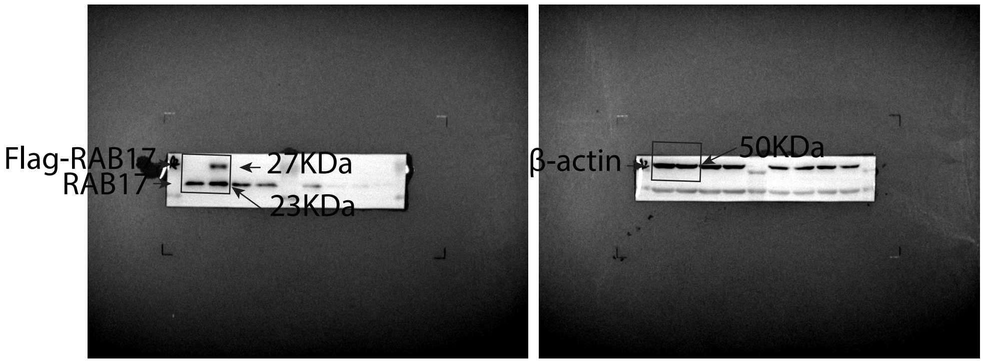

HSC-2

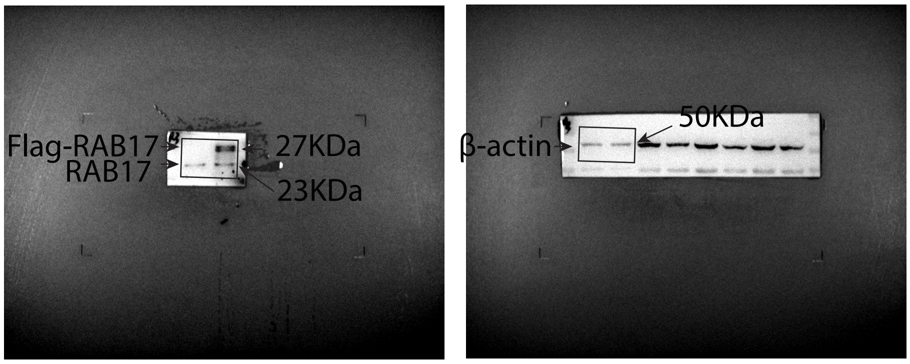

HSC-4

Figure 5E

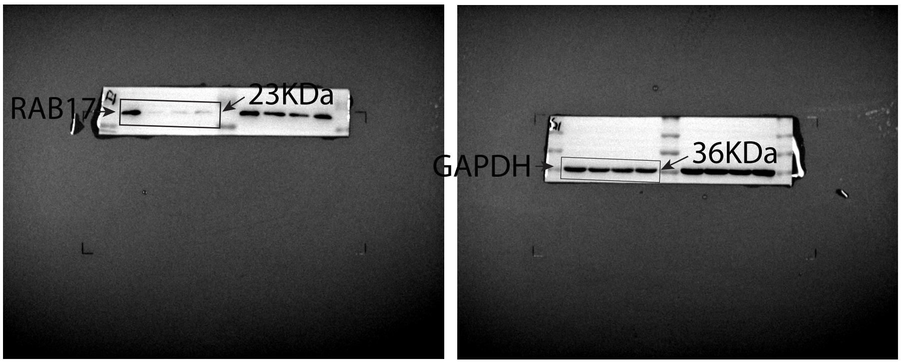

CAL27

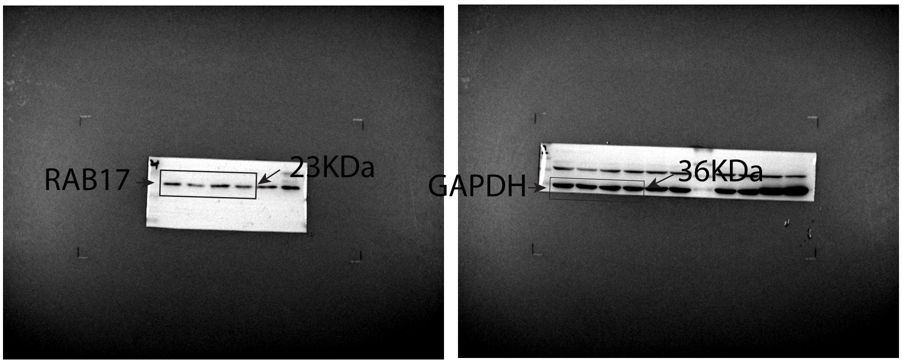

SCC9

Figure 6E-1

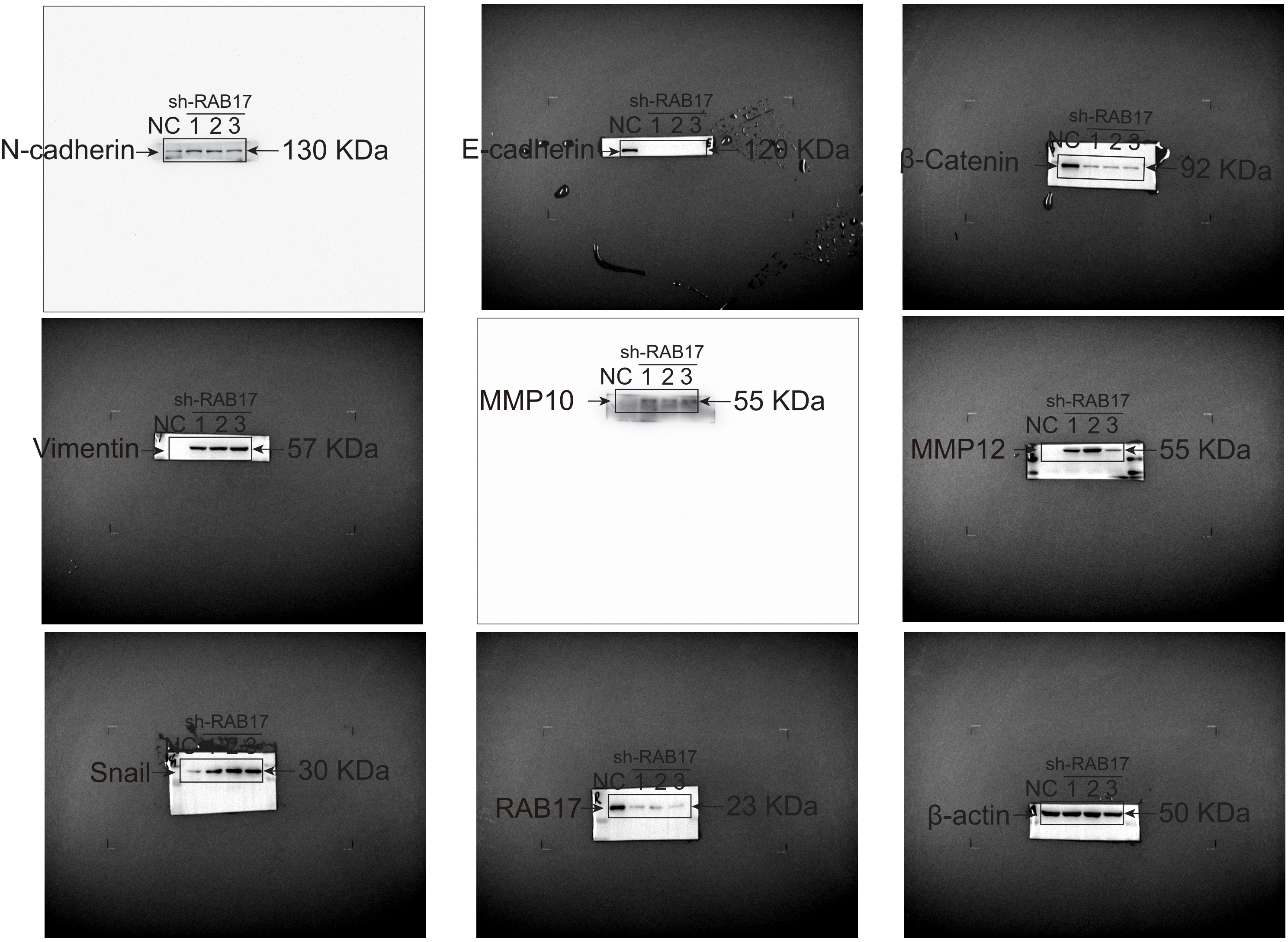

Figure 6E-2

CAL27 Cell lines

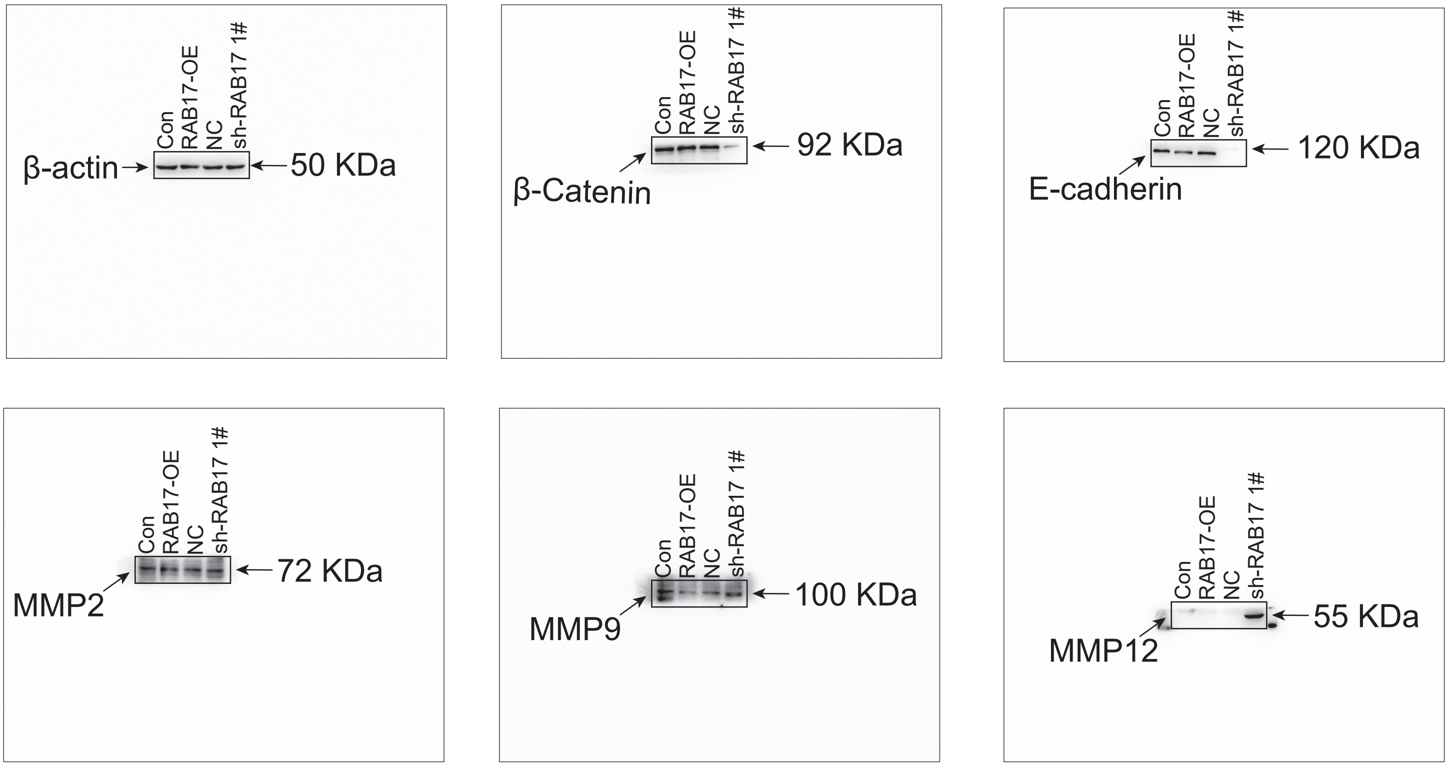

Figure 6E-2

CAL27 Cell lines 20230420

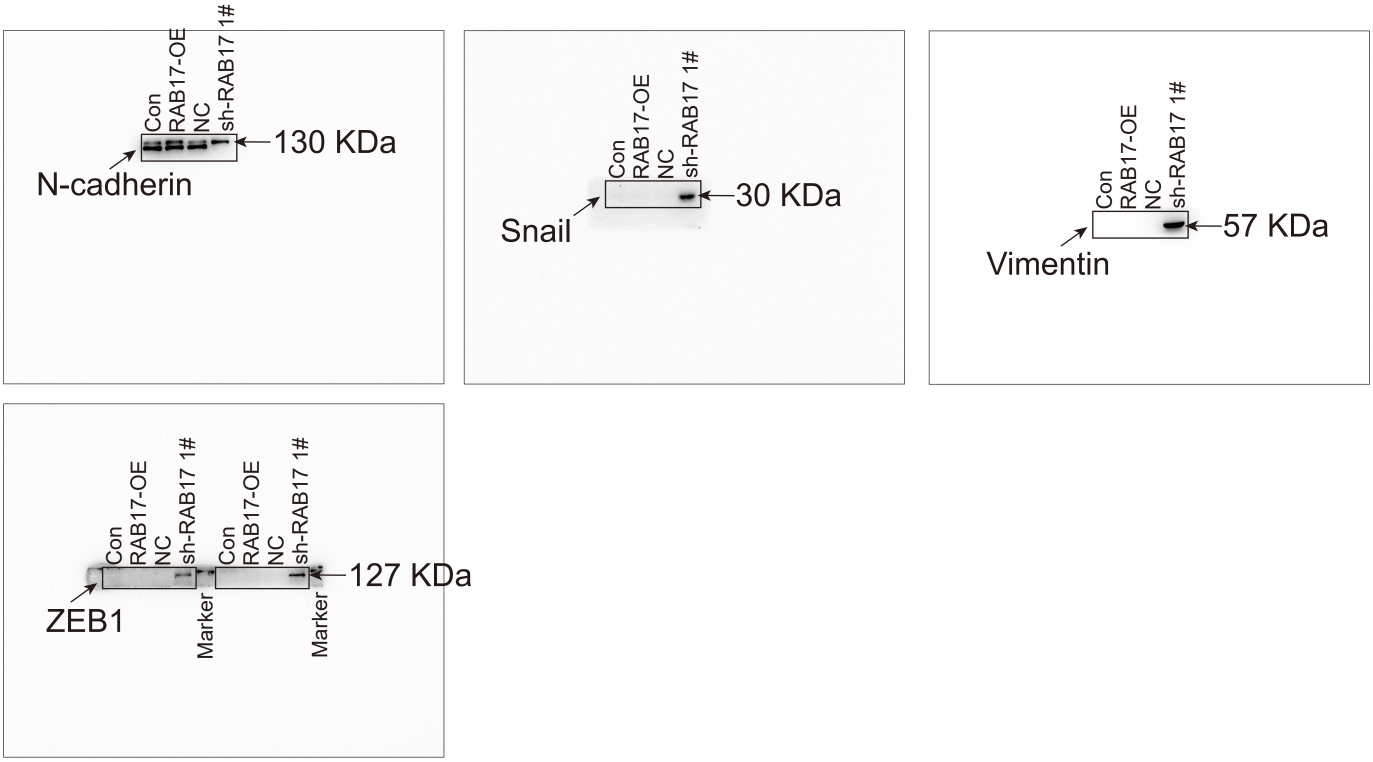

Figure 6E-3

CAL27 Cell lines 20230506

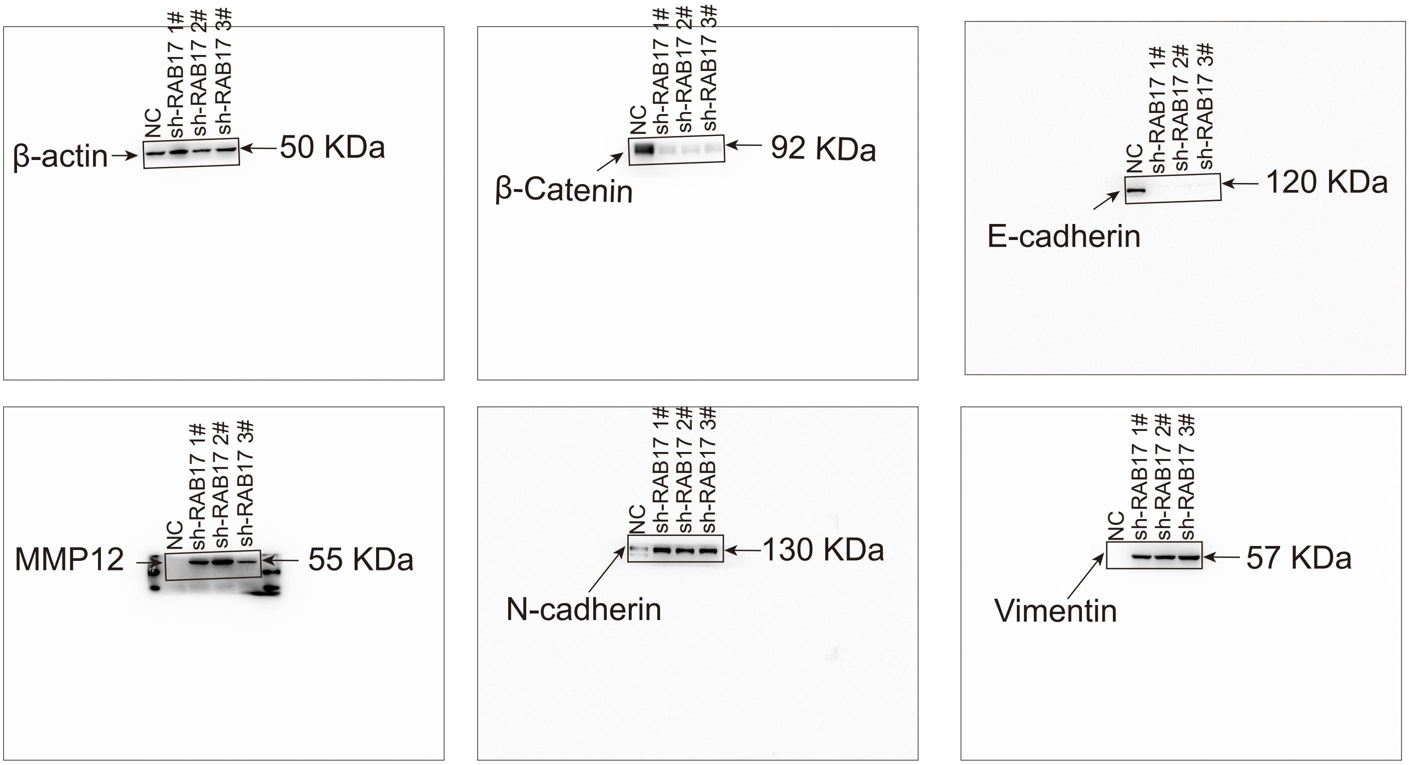

Figure 6E-4

20230602

the same results

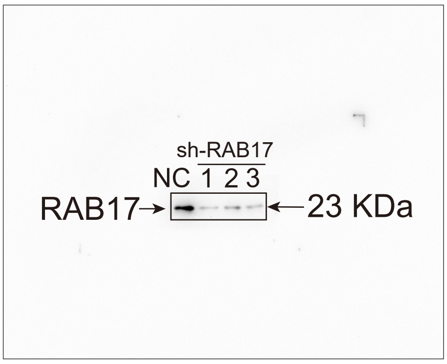

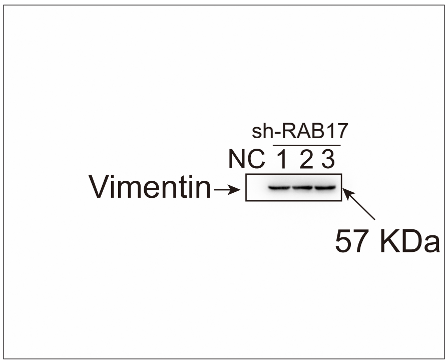

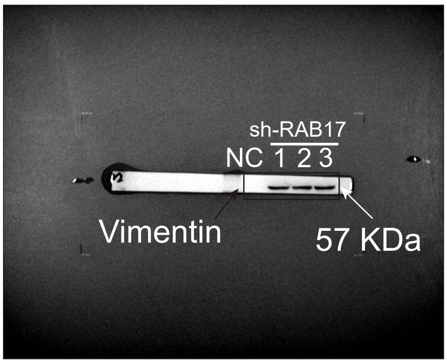

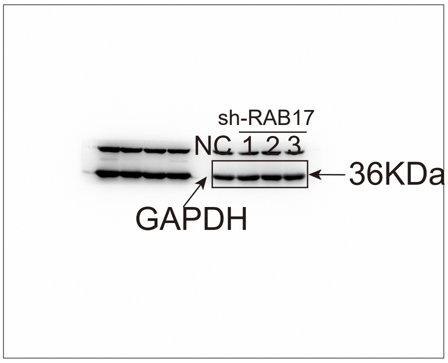

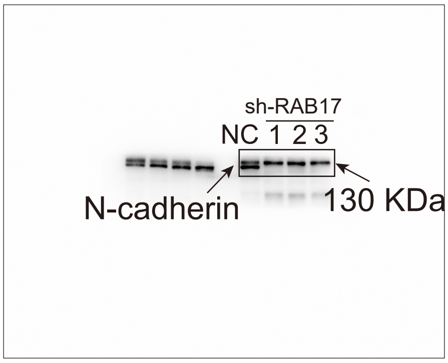

Figure 6F-1

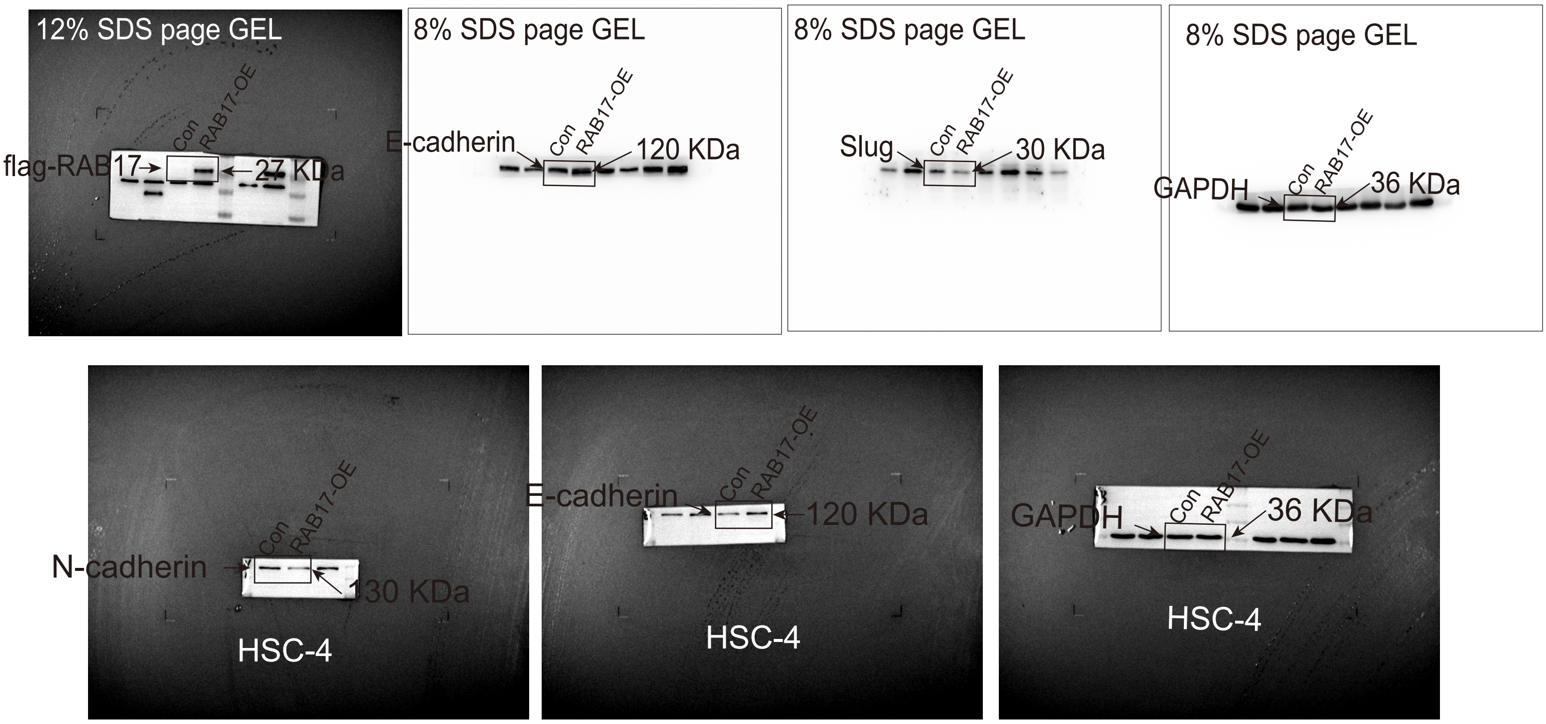

Since the two proteins are similar in size, we loaded and detected them separately.

Figure 6F-2 20230304-HSC-2

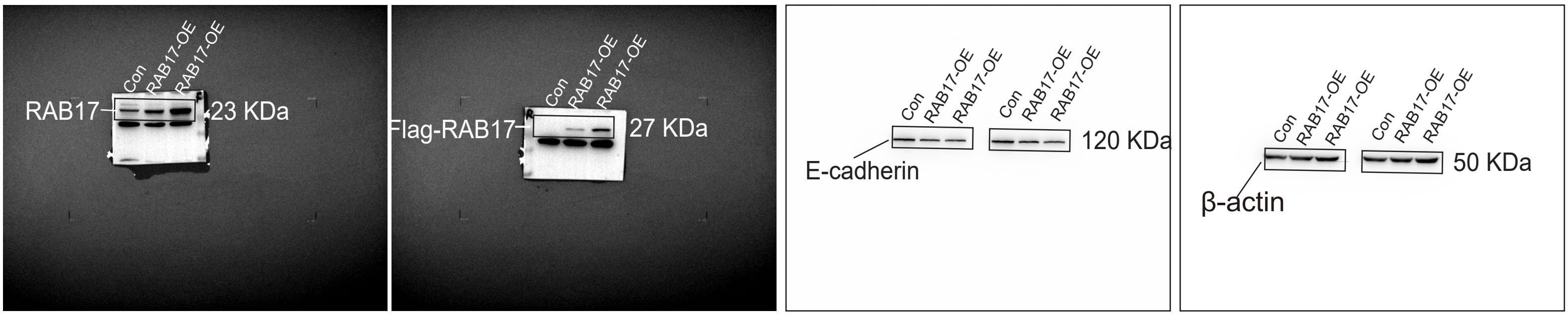

Figure 6F-3 20230908-HSC-2

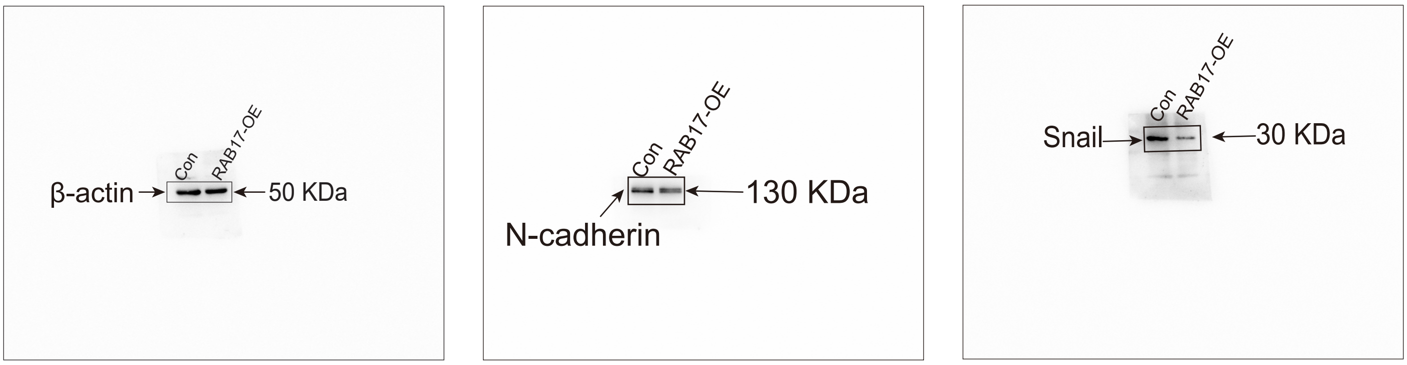

Figure 7C-1

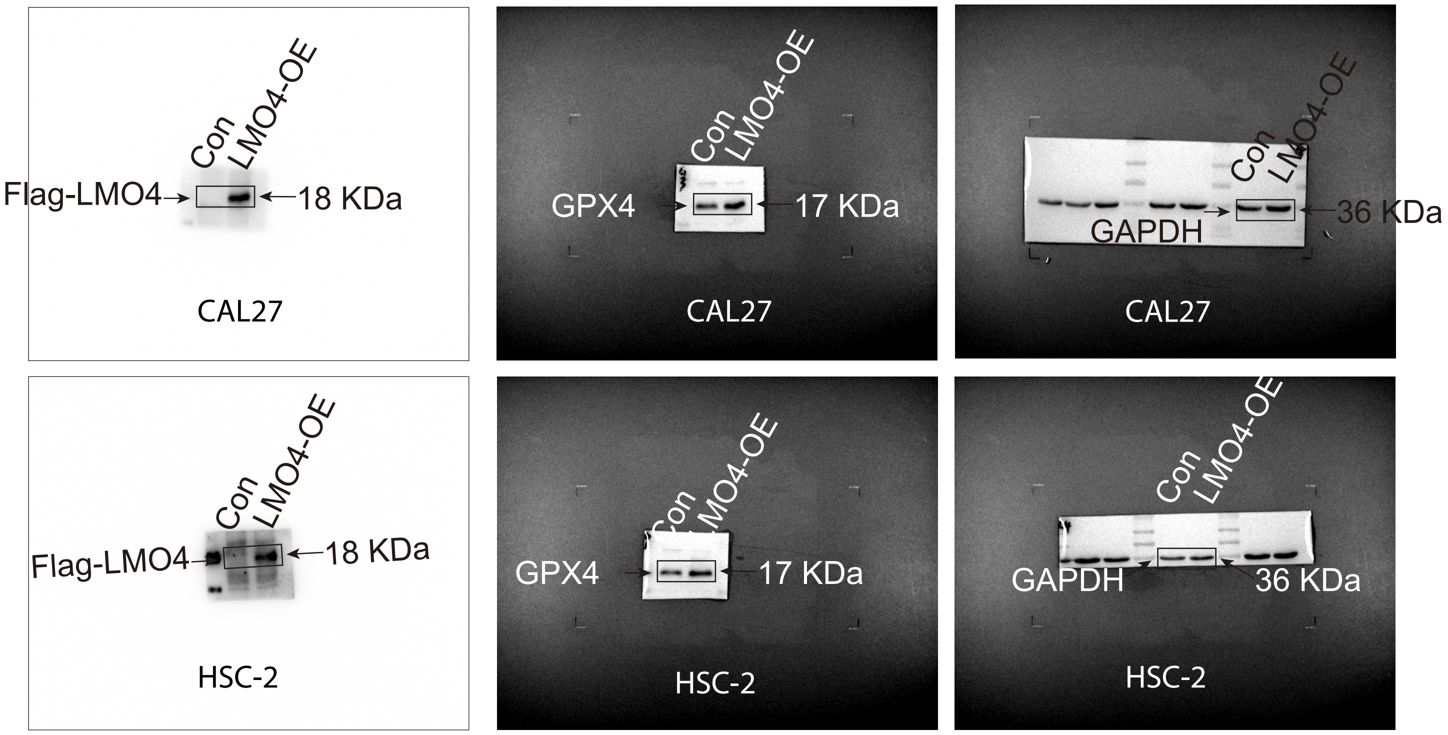

Figure 7C-2

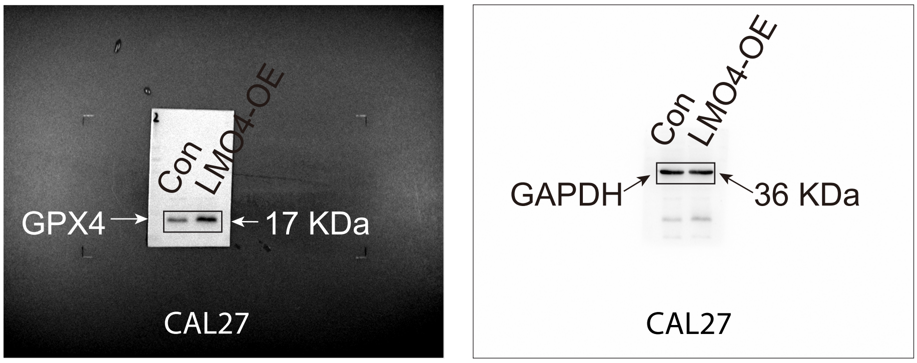

Figure 7C-3

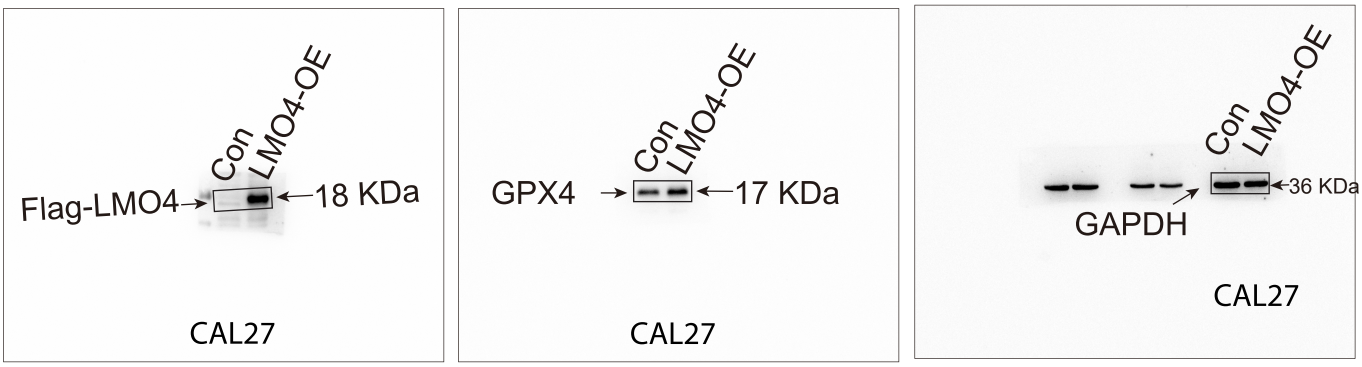

Figure 7C-4

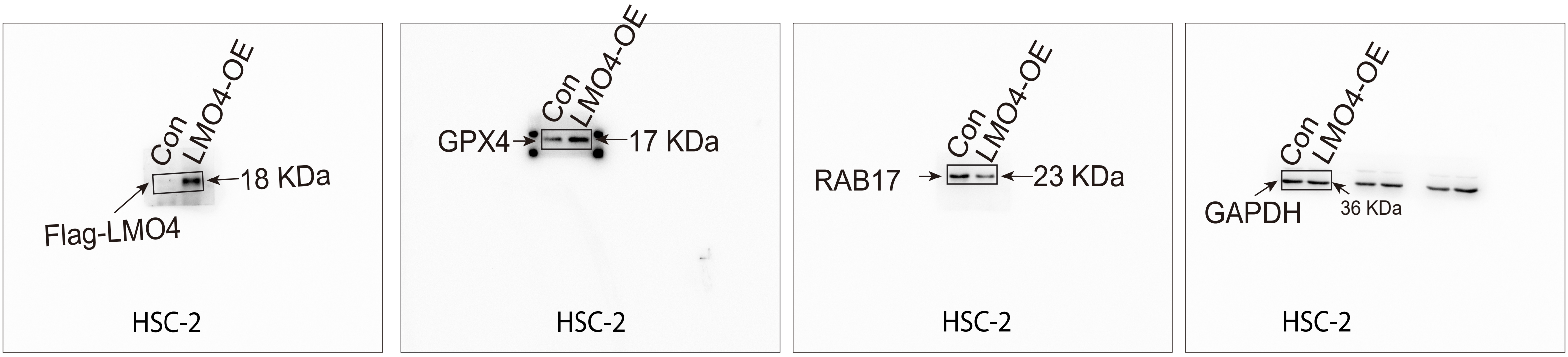

Figure 7D

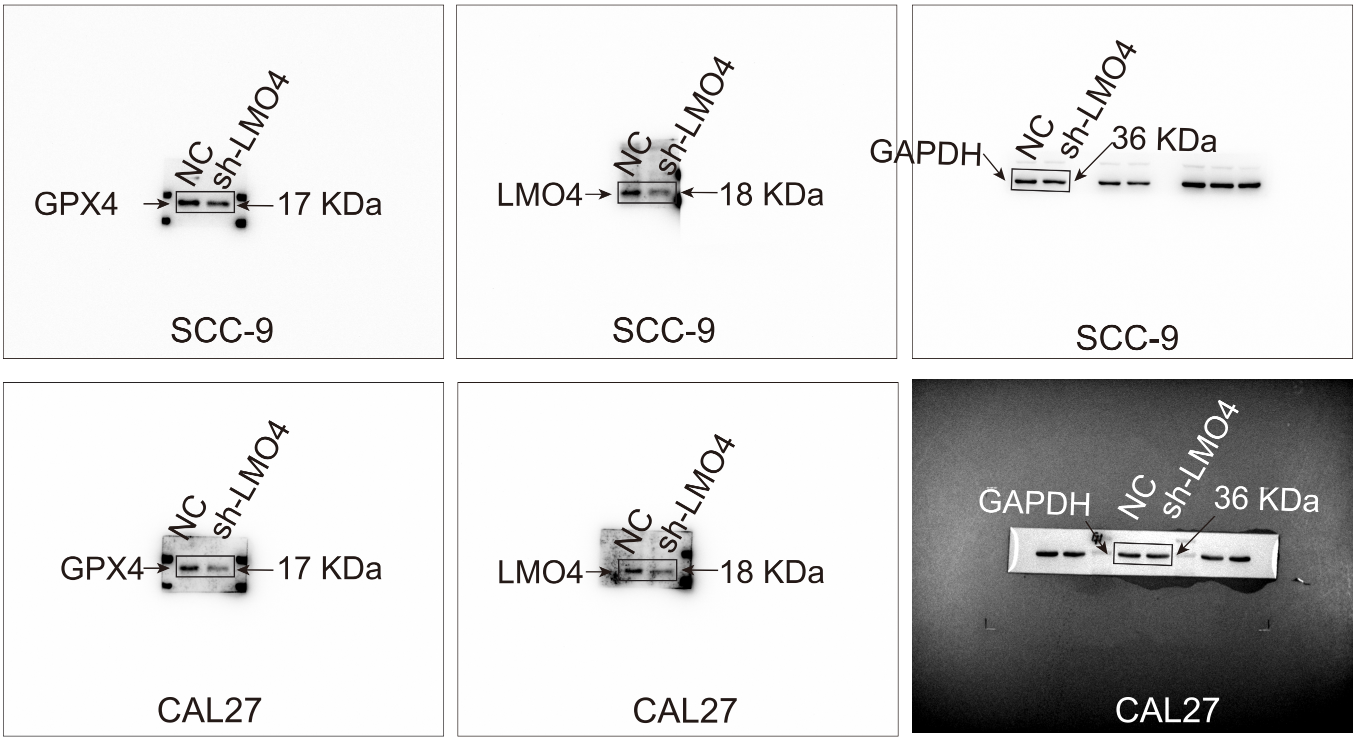

Figure 7E-1

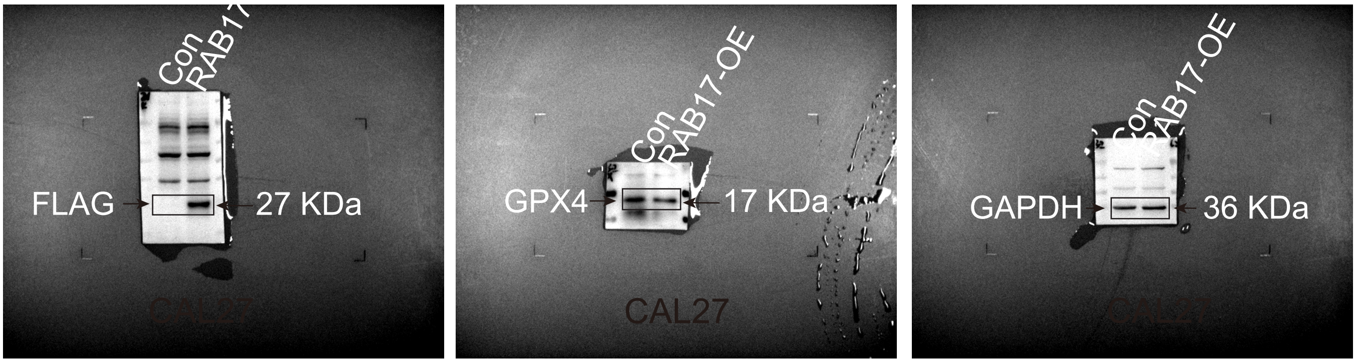

Figure 7E-2

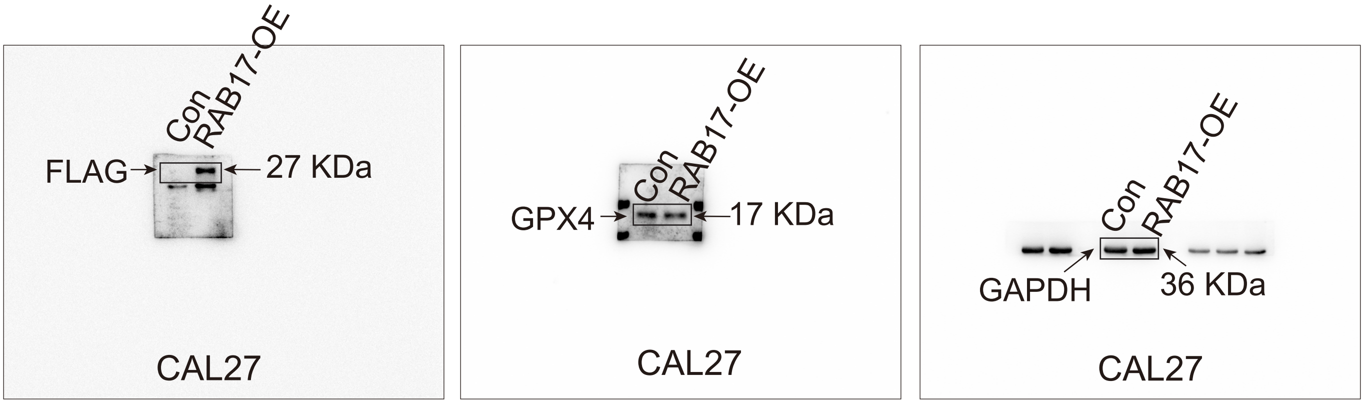

Figure 7F-1

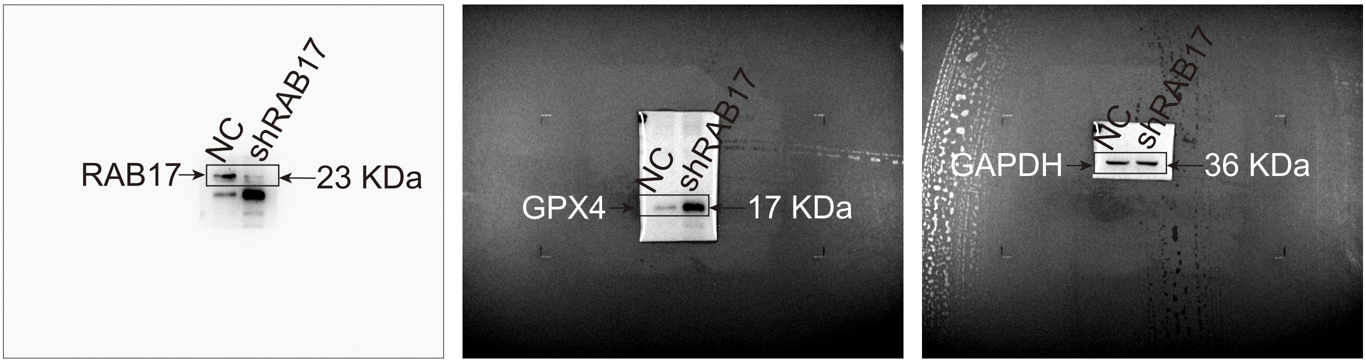

Figure 7F-2

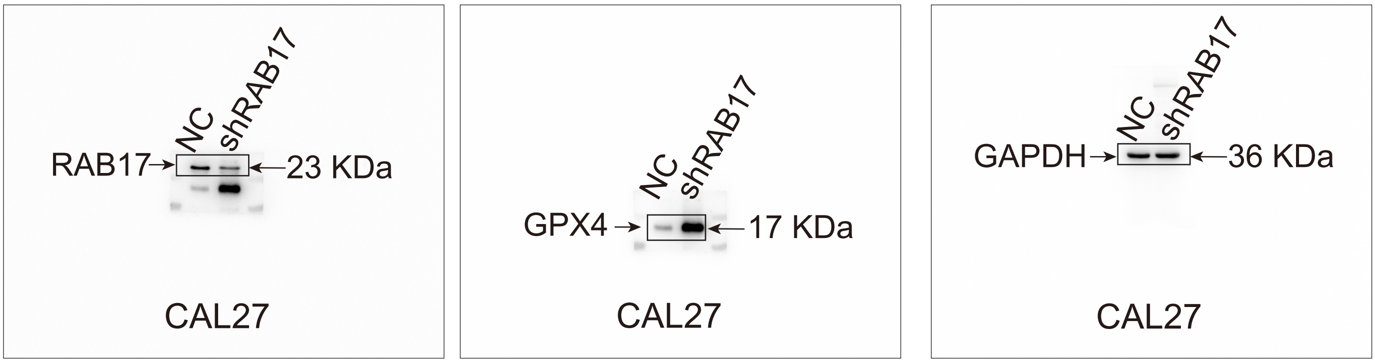

Figure 7F-3

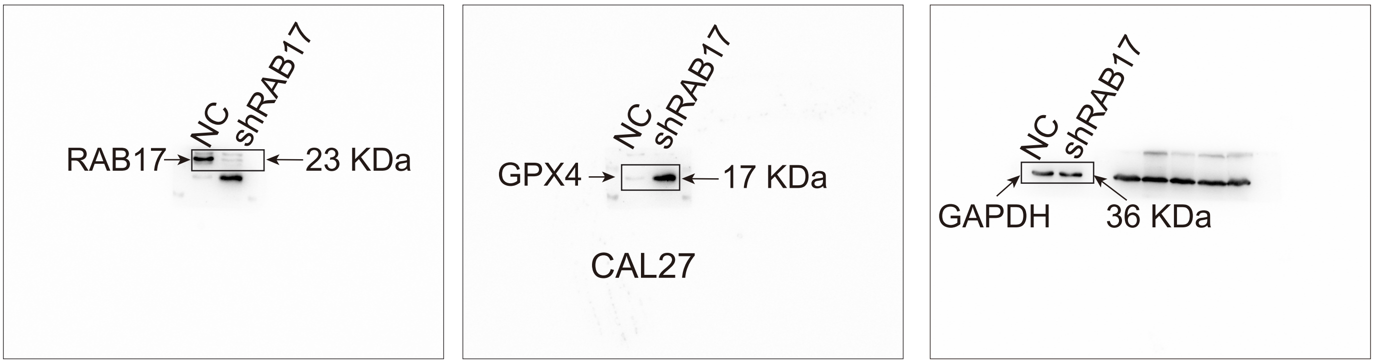

Figure 7G

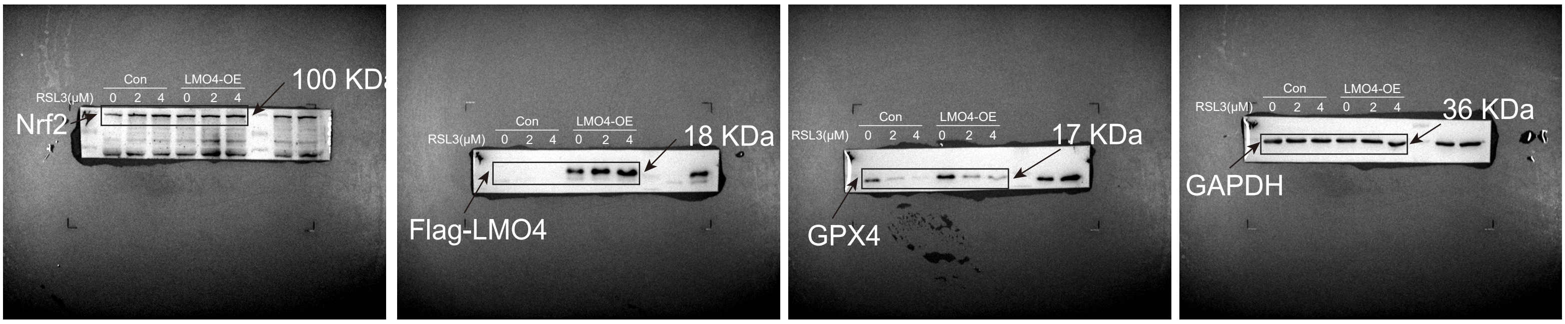

Figure 7H-1

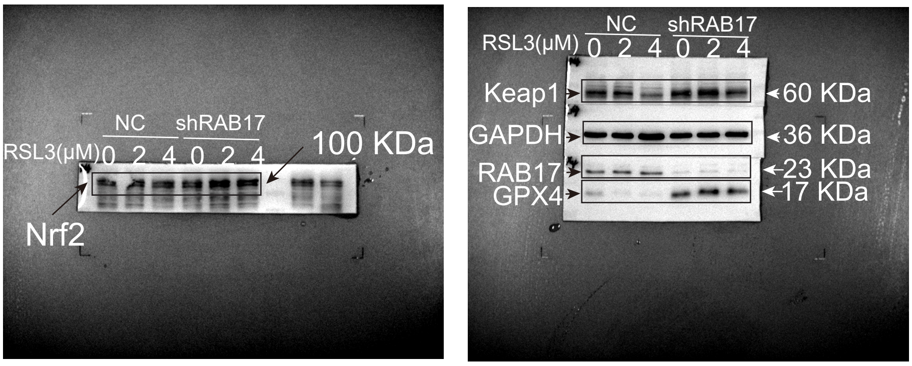

Figure 7H-2

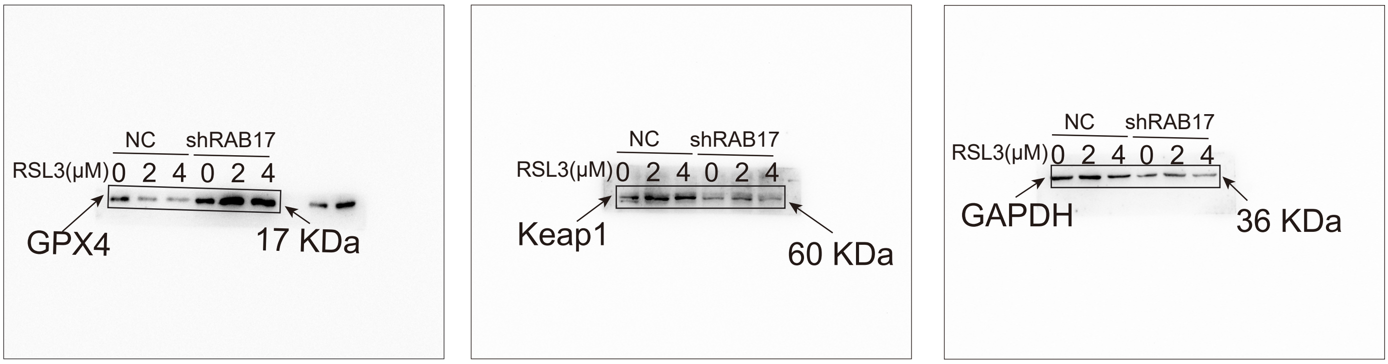

Figure 7H-3

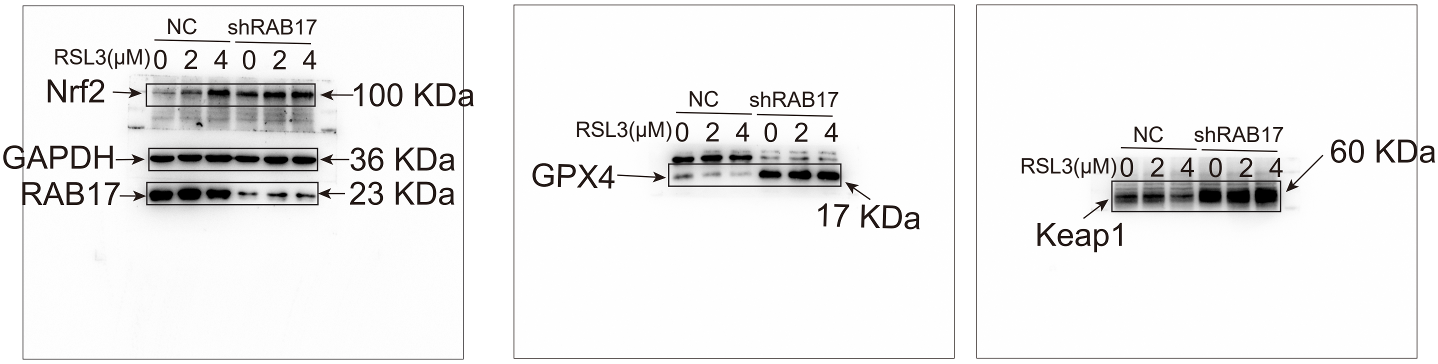

Figure 7I

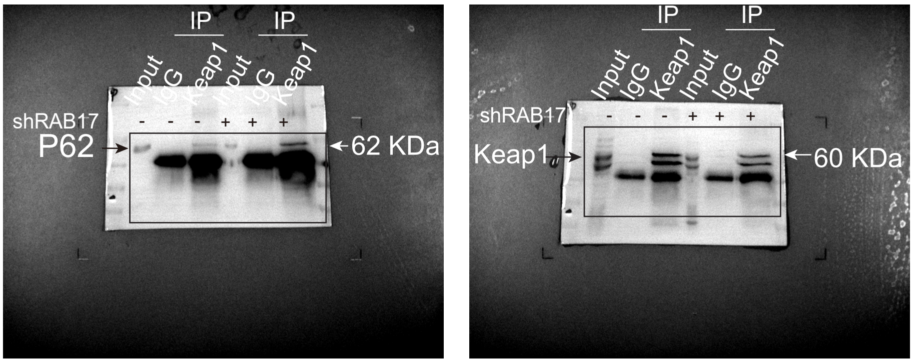

Supplement: Supplementary file 2 — Original Western blot images were cropped for presentation [file 41419_2025_8171_MOESM2_ESM.pdf]
